# Mechanistic dissection of the PD-L1:B7-1 co-inhibitory immune complex

**DOI:** 10.1371/journal.pone.0233578

**Published:** 2020-06-04

**Authors:** Sarah C. Garrett-Thomson, Aldo Massimi, Elena V. Fedorov, Jeffrey B. Bonanno, Lisa Scandiuzzi, Brandan Hillerich, Ronald D. Seidel, James D. Love, Scott J. Garforth, Chandan Guha, Steven C. Almo

**Affiliations:** 1 Department of Biochemistry, Albert Einstein College of Medicine, Bronx, New York, United States of America; 2 Department of Radiation Oncology, Albert Einstein College of Medicine, Bronx, New York, United States of America; 3 Cue BioPharma Inc., Cambridge, Massachusetts, United States of America; Duke University School of Medicine, UNITED STATES

## Abstract

The B7 family represents one of the best-studied subgroups within the Ig superfamily, yet new interactions continue to be discovered. However, this binding promiscuity represents a major challenge for defining the biological contribution of each specific interaction. We developed a strategy for addressing these challenges by combining cell microarray and high-throughput FACS methods to screen for promiscuous binding events, map binding interfaces, and generate functionally selective reagents. Applying this approach to the interactions of mPD-L1 with its receptor mPD-1 and its ligand mB7-1, we identified the binding interface of mB7-1 on mPD-L1 and as a result generated mPD-L1 mutants with binding selectivity for mB7-1 or mPD-1. Next, using a panel of mB7-1 mutants, we mapped the binding sites of mCTLA-4, mCD28 and mPD-L1. Surprisingly, the mPD-L1 binding site mapped to the dimer interface surface of mB7-1, placing it distal from the CTLA-4/CD28 recognition surface. Using two independent approaches, we demonstrated that mPD-L1 and mB7-1 bind in cis, consistent with recent reports from Chaudhri A et al. and Sugiura D et al. We further provide evidence that while CTLA-4 and CD28 do not directly compete with PD-L1 for binding to B7-1, they can disrupt the cis PD-L1:B7-1 complex by reorganizing B7-1 on the cell surface. These observations offer new functional insights into the regulatory mechanisms associated with this group of B7 family proteins and provide new tools to elucidate their function *in vitro* and *in vivo*.

## Introduction

The immunoglobulin superfamily (IgSF) includes approximately 400 proteins that comprise co-stimulatory molecules (i.e., CD28: B7-1), co-inhibitory molecules (i.e., PD-1: PD-L1, BTLN-2), cell adhesion molecules & cytoskeletal regulators (i.e., JAM-1, RAGE) and cytokine receptors (i.e., CSF-1 R). The spatial and temporal mechanisms by which these proteins modulate the immune system remain to be fully defined. Among the best characterized of these proteins are members of the CD28 receptor family (i.e., CD28, CTLA-4, ICOS and PD-1), a subset of the IgSF, which provides the principal signals for optimal T-cell function [1–3]. These signaling receptors share structural features and recognize related cell surface ligands from the B7 family within the IgSF (e.g., B7-1, B7-2, ICOSL, PD-L1 and PD-L2) with similar modes of interaction [4,5]. For example, the engagement of CD28 by B7-1 and B7-2 leads to T cell activation, while interaction of the same B7 ligands with CTLA-4, provides inhibitory signals needed to terminate the response. The inducible costimulatory receptor (ICOS) provides additional positive signals (i.e., co-stimulatory) upon binding ICOS-L, and engagement of PD-1 with either of its two B7-like ligands, PD-L1 and PD-L2, initiates further inhibitory pathways (i.e., co-inhibitory) [6].

Remarkably, even within the well-studied CD28/B7 families, additional interactions continue to be discovered. B7-1 has been demonstrated to bind PD-L1, resulting in context dependent bi-directional inhibitory signals that are not fully understood [7–13]. ICOS-L has been demonstrated to bind both CD28 and CTLA4, with the CD28:ICOS-L interaction being important for activation of both allogeneic and memory T-cells independent of the well-established ICOS receptor [8,13,14]. Furthermore PD-L2 was shown to interact with repulsive guidance molecule-b (RGMb), resulting in impaired respiratory tolerance [15,16]. These intersecting and competing interactions result in a complex network of signaling pathways, which represents a significant challenge for defining the contributions of individual interactions. Notably, recent reports demonstrate that hPD-L1 and hB7-1 bind in cis (on the same cell surface) [17,18], a result that further adds to this complexity and has implications for the interpretation of previous studies examining PD-L1:B7-1 associated processes [7,8,10,19,20].

Herein, we utilize two independent experimental approaches to confirm that mB7-1 and mPD-L1 interact in cis and additionally present data indicating that mCTLA-4 or mCD28 engagement alters the cell surface organization/presentation of mB7-1, which unexpectedly impacts the mPD-L1:mB7-1 cis interaction. Furthermore, we describe the application of two platforms, cell microarrays and high-throughput flow cytometry to identify the protein binding surfaces involved in the formation of the PD-L1:B7-1 complex. These studies demonstrate that PD-L1 utilizes overlapping, but non-identical recognition surfaces for the engagement of PD-1 and B7-1. We extend these findings by exploiting libraries of mB7-1 and mPD-L1 mutants to demonstrate that PD-L1 recognizes a surface overlapping with the B7-1 homodimer interface residing distal to the CTLA-4/CD28 binding surface. Importantly, these approaches supported the generation of set of unique PD-L1 and B7-1 variants with a range of biochemical properties, including selective receptor binding, for *in vitro* and *in vivo* analyses. These reagents provide important opportunities for dissecting and discovering additional mechanisms that impact normal physiology and pathologies.

Given their prominent role in regulating the immune response, it is not surprising that proteins within the CD28 family remain a primary focus for the development of targeted immunotherapies. Prime examples include ipilimumab (Yervoy™; Bristol Myers Squibb), a function blocking mAb against the CTLA-4 inhibitory receptor [21], and the function blocking mAbs nivolumab (OPDIVO^TM^; BMS) and pembrolizumab (KEYTRUDA™; MERCK) [22–24], which target the PD-1 inhibitory receptor. However, although these therapies are showing great promise and result in significant improvements to patient outcomes, they also can manifest severe deleterious side effects and elicit responses in roughly 2–40% of patients treated [25–28]. This clinical behavior highlights the importance of continued efforts to elaborate the complex immune regulatory mechanisms operating in normal physiology and disease. The approaches outlined herein are readily applicable to other receptor:ligand complexes in the B7 family and beyond, opening up possibilities for identifying better candidates for therapeutic interventions and generating more selective therapeutic modalities.

## Materials and methods

### Tissue culture and transient transfection

HEK 293 suspension cells were maintained in HEK Freestyle Media (Invitrogen) supplemented with Pen/Strep antibiotics and grown at 37 C in a humidified shaking platform incubator (Kuhner) with 5% CO_2_. For transfection, cells were pelleted at 500xg and resuspended in fresh media. For small-scale (1mL cells at 1x10^6^/mL) transient transfections performed in 24-well non-treated tissue culture plates, 2 μg Polyethylenimine (PEI) was added to 0.5 μg diluted plasmid DNA in a final volume of 100 μL. For large-scale transfections (600mL cells at 1x10^6^/mL) carried out in 2L baffled sterile shake flasks, 2 mg PEI was added to 400 μg diluted plasmid DNA.

### mPD-L1 and mB7-1 site-directed mutagenesis

All site-directed mutagenesis of mPD-L1 and mB7-1 was performed using high fidelity KOD polymerase, 2mM dNTPs and 4mM MgCl2. The template used for the mPD-L1 mutagenesis included the coding sequence for full-length mouse PD-L1 cloned between the SacI and BamHI sites of the Clontech N1 mCherry vector. For B7-1 the template used included the full-length native mouse mB7-1 coding sequence was cloned between the XhoI and SacII sites of the Clontech N1 mCherry vector by In Fusion (Clontech). Additional Information regarding the mutagenesis can be found in the supplemental information.

### Cell microarrays

#### Printing and transfection

Our cell microarray protocol was based on the method described by Sabatini and colleagues; the DNA plasmid concentration, percentage of gelatin, cell numbers plated, slides used and incubation times were all optimized for reproducibility in our system. A detailed description of our method can be found in the supplemental information.

#### Binding and analysis

Transfected cell microarray slides were washed three times with 1x PBS. Fc-fusion proteins of hCTLA-4, hCD200R, mPD-L1, mPD-1 and mB7-1 were purchased from R&D Systems. For microarray queries, 0.6 μg of Fc-fusion protein and 2.0 μg of Alexa 647 labeled Goat anti-mouse secondary antibody were premixed in a final volume of 300 μL 1x PBS and 0.2% BSA and incubated on transfected slides at RT for 30 minutes. Additional details can be found in the supplemental information.

### Microbead FACS binding assay

PD-L1 mCherry mutant constructs were transiently transfected into HEK 293S cells and subsequently queried with protein A microbeads (Milltenyi) pre-saturated with a 4:1 mixture of PD-1 Fc-fusion and FITC-Fc protein. A Fc protein to bead ratio of 5μg protein:10μL microbeads was utilized on the basis of a previous report from Genentech [15]. Please see the supplemental data for details regarding bead preparation, incubation with transfected cells and binding analysis.

### T-cell activation assay

Spleens were harvested from C57BL/6 mice and CD4^+^ T-cells isolated using mouse anti-CD4 microbeads (Milltenyi). The CD4^+^ T-cells were collected in complete RPMI media supplemented with 10% FBS, pen/strep antibiotics, 2mM L-glutamine and 0.1% BME. The cells (10^7^ / mL) were stained with 2.5 uM CFSE (Invitrogen) for 10 min at 37°C, washed two times with cold complete media and recounted. On the same day, 75,000 cells were plated per well in a 96-well TC plates in complete RPMI media and either left inactivated, activated with 33.3 nM (~ 5 μg /mL) anti-CD3, or activated with 33.3 nM anti-CD3 in the presence of a ~5-fold molar excess (174.3 nM) of either control Fc, WT PD-L1-Fc or mutant PD-L1 Fc proteins. Four days post activation, proliferation was determined by analyzing CSFE dilution. The data from each experiment were normalized to the control Fc population and a total of three independent experiments were averaged.

### B7-1 and PD-L1 co-transfection cis binding competition experiment

HEK 293 suspension cells were transiently transfected with WT mB7-1 mCherry or mB7-1 mutants in combination with WT mPD-L1 GFP. WT mPD-L1 GFP was also transfected alone as a control. For 1 mL transfection, 50 ng of each expression plasmid was used representing 1/10^th^ the total amount of DNA for transfection (pUC19 DNA was used as filler to reach 500 ng total) and 2 μg PEI. Transfection efficiencies were typically >60%. 100K cells from each transfection were incubated with either recombinant mPD-1 hIgG1 or mCTLA-4 hIgG1 protein (R&D systems) for 1 hour at room temperature. After binding, cells were washed 2X with PBS and 0.2% BSA and binding detected with anti-human (H+L) Alexa 647 secondary antibody (0.25 μg). Flow cytometric analysis was used to determine the percentage of mPD-L1 GFP expressing cells positive for Alexa 647 positive (PD-1 bound). Similarly, CTLA-4 binding was determined as Alexa 647 positive (CTLA-4 bound) as a percentage of mB7-1 mCherry expression (or mutant). PD-1 or CTLA-4 binding was normalized to that observed for cells expressing WT mPD-L1 alone or WT mB7-1 alone, respectively.

### Split nanoluciferase detection of cis bound PD-L1:B7-1

Split nanoluciferase constructs were generated by cloning full-length WT mB7-1, SER mB7-1, CYS mB7-1, WT mB7-2 and WT mPD-L1 into the NheI and XhoI of both the pBiT1.1-C (TK/LgBiT) and pBiT2.1-C (TK/SmBiT) vectors from Promega. For experiments, 1 mL of suspension HEK 293 cells were transfected with different combinations of SmBit and LgBit constructs (250 μg each) that were mixed and incubated with 2 μg PEI. Two days post transfection all transfected cell populations were counted twice and normalized to 1 x 10^6^ cells/ mL using Freestyle growth media (Invitrogen). For luminescence measurements, 50 μL of each normalized cell population were transferred to a 96-well half-well luminescence plate to which 12.5 μL of diluted Nano-Glo^®^ live cell substrate (Promega) was added. Cells were incubated with substrate for 10 minutes and then immediately read on an Envision plate reader (Perkin Elmer) to detect luminescence using a 5 sec integration time. Details regarding the competition experiments using the split nanoluciferase can be found in the supplemental information.

Additional methods are described in the Supplemental Methods.

## Results and discussion

### Overall results

We confirmed the previously reported mPD-L1:mB7-1 interaction using cell microarray- and high-throughput flow cytometric-based approaches, and employed these same strategies to systematically evaluate libraries of point mutants to define the residues contributing to the binding interface. Using full-length mouse B7-1 and PD-L1 constructs, complementary cell-based approaches involving reconstitution of split reporter proteins corroborated the recent report that the IgV domains of human B7-1 and PD-L1 engage in a cis-interaction on the plasma membrane of the same cell. *In vitro* competition assays using recombinant proteins demonstrated that mPD-1 directly competes with mB7-1 for binding to mPD-L1, while mCTLA-4 and mCD28 do not directly compete with mPD-L1 for binding to mB7-1. In contrast, in cell-based experiments, we observed mCTLA-4- and mCD28-dependent reorganization of cell surface expressed mB7-1 and mB7-2 and demonstrate that this reorganization in turn affects cis mPD-L1:mB7-1 complex formation.

### Cell microarray analysis

#### Platform validation

**Fig 1:**
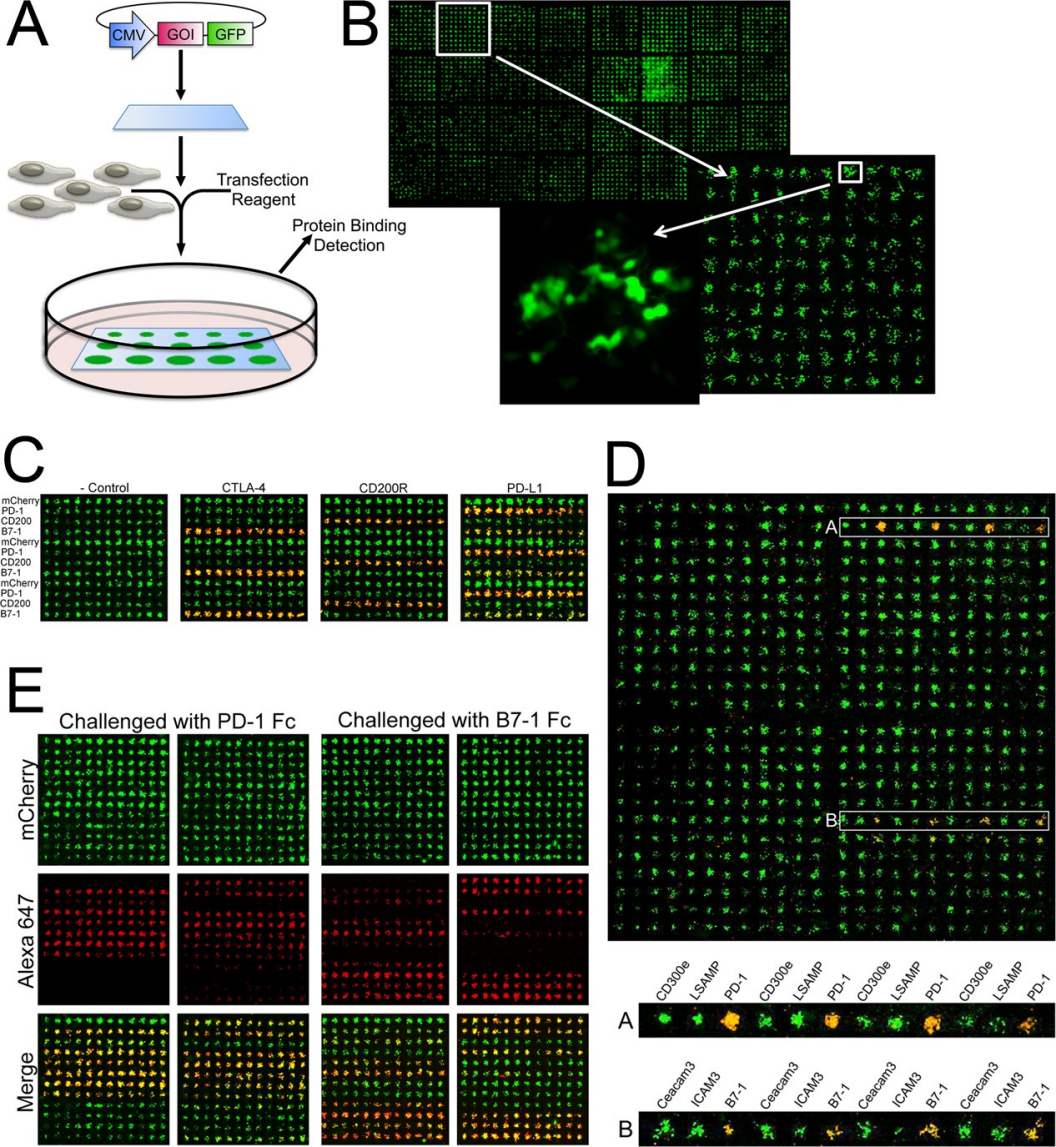
The use of the cell microarray platform to identify secreted protein interactions. A) Schematic for generating cell microarrays. B) For illustration, a GFP expression construct was “pinned” onto a glass surface to create an expression array. At high magnification individually transfected cells can be detected with each spot containing 50–80 cells. C) Slides were printed with alternating rows of plasmid DNA encoding mCherry fusion constructs of PD-1, CD200, B7-1 or mCherry alone. Printed slides were transfected and subsequently treated with Fc-fusions (~100nM) of IgG control, CTLA-4, CD200R and PD-L1. Fc-fusions were pre-incubated with Cy7 secondary antibody (~200 nM). for detection. For each array, significant binding of the Fc-fusion is detected for only those rows where its cognate receptor or ligand is present. D) Slides were printed with expression constructs for 144 human genes in the Ig superfamily. Each construct was printed in 4 replicates across a row resulting in a total array of 4 x 144 spots. This cell array was treated with 100 nM recombinant PD-L1-Fc pre-incubated with 200 nM Alexa 647 labeled anti-human IgG, washed and fixed with 4% formaldehyde (pseudo-colored red, single channel not shown). The overlaid green and red pseudo-colored images appear yellow/orange where binding is observed due to the merging of the green and red fluorescence signals. The rows labeled A and B contain the two known binding targets of PD-L1, PD-1 (A) and B7-1 (B). 10x magnification of the rows highlighted in clearly shows the positive signal observed for the PD-L1:PD-1 and PD-L1:B7-1 interactions as compared to the signal observed from the surrounding spots. E) Slides were printed with rows of plasmid (24 rows total) encoding (alternating between the right and left grids) WT PD-L1, mCherry (-control), D28A, D28R, D49A, D49R, V54A, V54R, Y56A, Y56D, Q66A, Q66D, E72A, E72R, G119D, G119R, G120D, D122A, Y123A, Y123R, K124A, K124D, R125A, R125D. The data shows two representative slides (2 grids/slide = 24 rows = mutants listed) queried with either PD-1 or B7-1 Fc-fusion protein detected with an anti-human Alexa 647 antibody.

To evaluate the murine PD-L1:B7-1 interaction, we first employed the cell microarray technology introduced by Sabatini and colleagues [29,30]. This approach enables the presentation of large numbers of wild type or mutant cell surface molecules in the context of live host cells in a precisely arrayed format. Briefly, each expression construct is individually “pinned” onto a glass surface to create an expression array of library molecules (Fig 1A and 1B). Mammalian cells, when plated over the printed cDNAs in the presence of transfection reagent (e.g., lipid-based reagent), become transfected, resulting in a living cell array, with each individual cluster of ~50–80 cells expressing a single member of the library. Cells growing between the printed cDNAs are not transfected and remain “black”; a small representative grid of cells expressing cytoplasmic GFP is shown in Fig 1. If the cDNAs code for cell surface proteins, these expression arrays can be queried with purified fluorescently tagged query proteins (e.g., Fc fusion proteins). Positive interactions are scored as a function of fluorescence intensity after washing to remove unbound ligand. As each construct is “pinned” at a known position in the microarray, a positive “hit” can immediately be correlated with its interacting partner(s) and the level of promiscuity readily determined.

Ligation Independent Cloning (LIC) [31] was used to generate a library of expression constructs for ~200 members of the human Ig and TNFR superfamilies, in which each gene of interest is followed by a transmembrane anchor and covalently fused at its C-terminus (type-I membrane proteins) to a cytoplasmically-localized mCherry expression reporter (S1 Fig). The overall design for the membrane display vector and the strategy for Fc-fusion production is presented in S2A and S2B Fig, respectively.

The cell microarray platform was initially validated using the known murine PD-L1:PD-1, CTLA-4:B7-1 and CD200R:CD200 interactions. Live cell microarrays consisting of alternating rows of cells expressing mCherry fusions of murine PD-1, B7-1, CD200 or mCherry alone (Fig 1C; cells transfected on the chips are pseudocolored GREEN) where queried with Alexa 647-bound bivalent Ig-fusion (i.e., RED Fc-fusions) constructs of the murine CTLA-4, CD200R or PD-L1 ectodomain. These experiments validate our implementation of cell microarray technology, highlight the signal to noise and underscore the ability to correctly identify cognate receptor:ligand interactions.

#### Microarray analysis of mPD-L1 interactions

To evaluate mPD-L1 interactions, mPD-L1 Fc fusion protein was used to query a cell microarray array presenting 144 members of the human IgSF. These results clearly demonstrate mPD-L1 binding to both mPD-1 and mB7-1(Fig 1D), highlighting the suitability of this platform to identify multiple binding partners and confirming the PD-L1:B7-1 interaction with the murine orthologs. Quantification of the fluorescence intensity of bound PD-L1 showed an average signal of 923 RFU for PD-1 binding (16-fold above background) compared to 330 RFU for B7-1 binding (5.5-fold above background), which is consistent with the higher affinity reported for PD-1 binding compared to B7-1 [8].

#### Biochemical and mechanistic dissection of the mPD-L1:mB7-1 interaction by microarray analysis

To generate more selective PD-L1 reagents, the X-ray structure of the PD-L1:PD-1 complex [32] was used as the basis to identify 36 solvent accessible residues within the PD-L1 Ig variable domain for mutagenesis. Each residue was changed to an alanine, arginine and glutamic acid in order to sample a range of side chain physico-chemical properties. The cell microarray platform was used to present wild type and mutant PD-L1 constructs, which were queried with mPD-1 or mB7-1 Fc-fusion protein. These experiments identified mutants that affected only mPD-1 binding (D122A, Y123A, Y123R, K124A, K124D, R125A, R125D), only mB7-1 binding (Y56A, Y56D, E72R, G119D, G120D) or both (L53R, G119R, A121R) (Fig 1E and S1 Table). However, consistent quantification of the B7-1:PD-1 binding interaction proved difficult using the cell microarrays because **1)** the lower affinity of B7-1 for PD-L1 reduced the signal to noise compared to those queried with PD-1; **2)** complete loss of binding was easily identified, but modest reductions in binding were often more variable; **3)** inherent slide-to-slide variability associated with independently printed, transfected and treated slides resulted in signal to noise variations that precluded more quantitative comparisons.

### Validation of mPD-L1 selectivity mutants by FACS analysis

**Fig 2:**
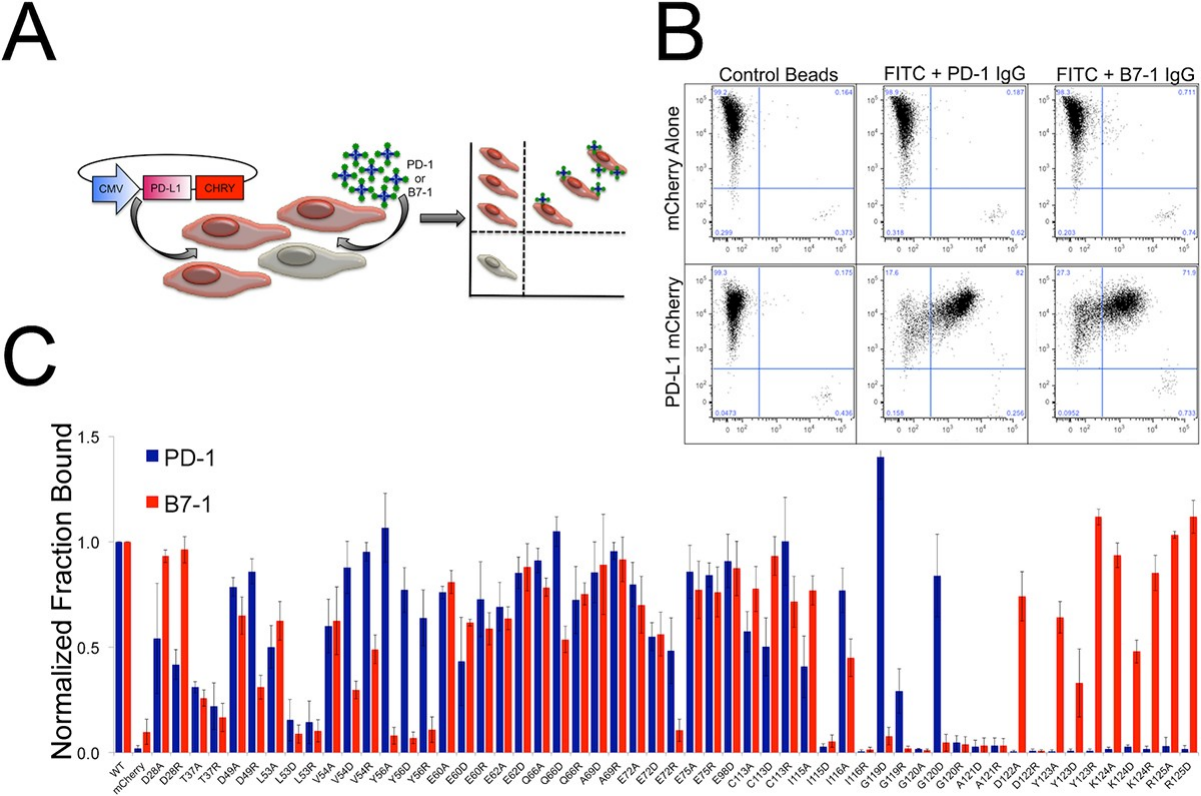
Screening PD-L1 mutants using a high-throughput microbead binding FACS assay. A) Schematic of the microbead FACS binding assay. B) Representative control microbead experiment. Cells expressing either mCherry alone (-control) or PD-L1 mCherry were queried with microbeads conjugated with control Fc, PD-1 Fc or B7-1 Fc fusion protein. The FACS data was gated for all live cells and shows binding of both PD-1 and B7-1 coated microbeads (upper right quadrant) to cells expressing wild type PD-L1. C) FACS microbead binding data for a panel of 54 PD-L1 mutants. Data shows the fraction of mCherry positive cells (PD-L1 expressing) bound to microbeads coated in either PD-1 (Blue) or B7-1 (Red) with binding normalized to wild type. PD-1 and B7-1 binding was done in parallel triplicate experiments with error bars representing the standard deviation.

To more quantitatively evaluate the binding characteristics of PD-L1 mutants, we implemented a high-throughput fluorescence activated cell sorting (FACS) assay, which enables the interrogation of 400 samples per hour. This FACs platform affords an enhanced dynamic range compared to cell microarrays and employs a modified strategy for presentation of the query protein. While bivalent Ig-fusions, as used in the microarray platform, are effective for the identification of interactions with high or moderate affinities, weaker interactions might be missed. To support detection of the wide range of apparent affinities anticipated in the library of PD-L1 mutants, we exploited the higher valency afforded by magnetic microbead capture and presentation (Fig 2A). For example, probing the microarray presenting PD-L1 required higher concentrations of B7-1 Fc than PD-1 Fc, resulting in greater background signal. The increased dynamic range of the FACS microbeads assay (on average 200-fold above background compared to 5-20-fold for the cell microarrays) is likely due, in part, to the reduction in background caused by non-specific binding. This effect is likely the consequence of the higher avidity associated with bead-presented query molecules, which allows lower amounts of protein to be used to query the cells, and the fact that no secondary antibody is used in the microbead assay. The microbead assay has the added benefit of not requiring any wash steps, which minimizes loss of bound sample and provides a more direct measure of protein binding.

**Fig 3:**
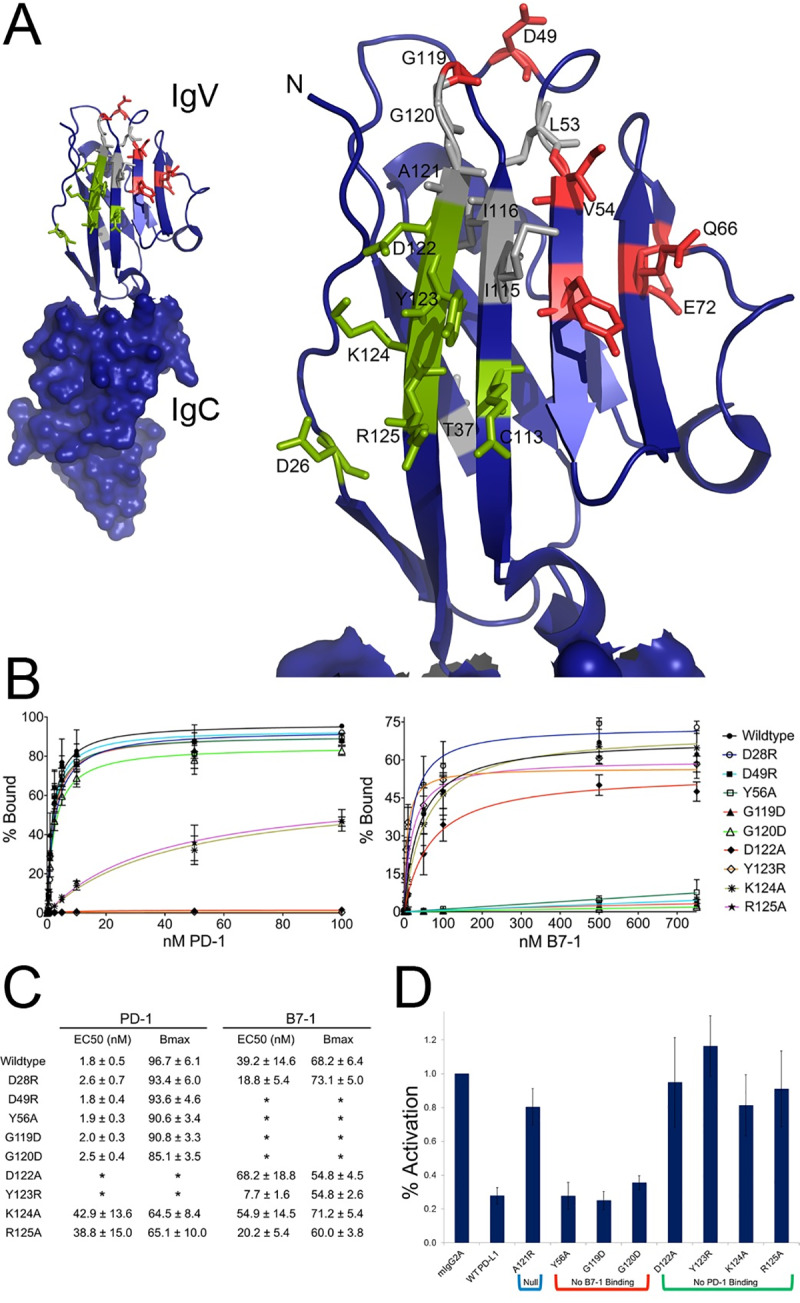
Characterization of PD-L1 mutants with altered binding to PD-1 or B7-1. A) The crystal structure of the PD-1: PD-L1 complex (PDB: 3SBW) showing just the PD-L1 IgC and IgV domains. The IgV domain was enlarged and residue that when mutated resulted in altered binding are labeled and colored accordingly, green = PD-1 binding affected, red = B7-1 binding affected, gray = both PD-1 and B7-1 binding affected. B) Data obtained from FACS titration experiments in which cells expressing either wild type PD-L1 or a mutant were titrated with increasing concentrations of recombinant PD-1 or B7-1 Fc-fusion protein. Binding was detected using an anti-mouse Alexa 488 secondary antibody. Data points show the average of three independent experiments with error bars showing the standard deviation. Curves show the fit of the data to a single-site binding model. C) Table of EC_50_ and B_max_ values obtained from the FACS titration experiments in B. Here, EC50 refers to the effective concentration at which 50% binding was observed. Stars denote those titrations for which binding was so low (baseline) that the data could not be fit. D) Data shows the fraction of CSFE labeled CD4^+^ T-cells isolated from C57BL/6 mice activated after 4 days of stimulation with anti-CD3 in the presence of isotype control, wild type or mutant PD-L1 Fc-fusion protein. Activation was normalized to isotype control and represents three independent experiments.

Briefly, HEK293 cell lines were individually transfected with 55 different surface displayed mutant mPD-L1-mCherry fusions. These cells were probed by flow cytometry for their ability to bind FITC-decorated microbeads loaded with either wild type mPD-1 Ig-fusion or wild type mB7-1 Ig-fusion proteins (Fig 2B). Importantly, it is unlikely that these mutations caused global changes to the structure or stability of mPD-L1, as based on the percent and intensity of mCherry fluorescence, the transient protein expression levels were similar to wild type for all mutants examined (S3 Fig). Furthermore, fluorescence microscopy of the wild-type and mutant mPD-L1 variants showed correct plasma membrane localization of the C-terminal mCherry fusion proteins, consistent with the mutant proteins being correctly processed, folded and presented on the plasma membrane (TOP S4 Fig). In addition a subset of selectivity mutants that were identified in our screen exhibited monoclonal antibody binding comparable to wild-type PD-L1, suggesting these mutants are cells surface expressed and folded (BOTTOM S4 Fig). These studies resulted in the identification of mPD-L1 mutants with reduced binding to mPD-1 (D122A, Y123R, Y123A, K124A, K124D, K124R, R125A, R125D), or mB7-1 (D49R, V54D, V54R, Y56A, Y56D, Y56R, Q66D, E72R, G119D, G120D), or both mPD-1 and mB7-1 (L53D, L53R, I115D, I116R, G119R, G120A, G120R, A121D, A121R, D122R) (Fig 3C, S5 Fig and S2 Table). Mapping these residues onto the crystal structure of the PD-L1:PD-1 complex suggests that overlapping but distinct PD-L1 surfaces are responsible for PD-1 and B7-1 binding (Fig 3A and S6 Fig). The results obtained by flow cytometry (S2 Table) are in close agreement with those obtained from our initial cell microarray experiments (S2 Table). We also tested mPD-1 and mB7-1 binding against a set of 65 PD-L1 variants harboring mutations in the IgC domain. No effect on mPD-1 binding was observed for any of the mutants examined. A few mPD-L1 mutants including; S142A, E163A, E163R, M188A and Q214A showed modest effects on mB7-1 binding (~50% of wild-type B7-1 binding). Only one mPD-L1 IgC mutant, T185D, completely lost binding to mB7-1 (S7 Fig).

### Characterization of mPD-L1 mutant binding interactions

As the output of FACS analysis is mean fluorescence intensity per event (i.e., per cell), relative binding affinities of individual panel members can be assessed by comparison to wild type. This approach was used to further evaluate the binding characteristics of a selected panel of mPD-L1 mutants with altered binding to mPD-1, mB7-1 or both. Transiently transfected HEK cells expressing cell surface presented mPD-L1 wild type or mutant proteins were titrated with soluble mPD-1 or mB7-1 Fc-fusion proteins and binding detected using a fixed concentration of anti-mouse IgG H + L Alexa 488 secondary antibody. Saturation binding curves were collected in triplicate, averaged, and fit to a single-site binding model (Fig 3B), which revealed the altered binding properties of several of the mutants (e.g., EC_50_ and B_max_) (Fig 3C). It is important to note that the equilibrium dissociation constant (K_d_) of the mPD-L1:mB7-1 interaction was previously reported to be 1.6 μM, which is about three times weaker than that of the mPD-L1:mPD-1 interaction (K_d_ of 0.59 μM) [8,10].

### mPD-1 competes with mB7-1 for binding to mPD-L1 on beads

Our mutagenesis data are consistent with overlapping binding surfaces for mPD-1 and mB7-1 on mPD-L1, suggesting that mPD-1 and mB7-1 should compete with one another for binding to PD-L1. This hypothesis was directly tested with a bead-based FACS competition assay utilizing mB7-1 and mPD-1 Fc-fusion proteins harboring two different Fc fusion isotypes. Beads coated with PD-L1 (mIgG2a) and were incubated with a fixed saturating concentration of mB7-1 (hIgG1), and titrated with mPD-1 (mIgG2a) or mIgG2a. The concentration of mB7-1 required to saturate the beads was determined empirically by incubating a fixed amount of mPD-L1 coated beads with increasing concentrations of mB7-1. Competition was monitored using an anti-human Alexa 488 secondary antibody. S8 Fig shows that mPD-1 effectively competes with soluble mB7-1 in a concentration dependent manner for binding to mPD-L1 presented on beads.

### Activity of mPD-L1 mutants in a T-cell proliferation assay

We optimized high-throughput transient transfection of HEK 293 cells in 24-well suspension tissue culture plates for the production of recombinant secreted Fc-fusion proteins in amounts consistent with screening. Utilizing this method, we purified Fc-fusion proteins representing a subset of the mPD-L1 mutants with altered binding characteristics (S9 Fig). Following small-scale nickel purification of the mPD-L1 proteins, analytical gel filtration demonstrated that the selected mutants possessed solution properties (i.e., aggregation state) similar to wild type protein (S10 Fig and S11 Fig). Prior to use in T cell proliferation studies, the quality of each mutant protein was evaluated by FACS analysis for binding to HEK cells expressing surface-resident mPD-1 or mB7-1 (GFP fusions) to confirm that the soluble reagents behaved as expected (e.g., mPD-L1_Y56A_Fc binds to cells expressing mPD-1, but not to those expressing mB7-1 (S8 Fig)).

To characterize the biological activity of the functionally dissected mPD-L1 mutants, we utilized an *in vitro* T-cell activation assay involving plate-bound anti-CD3 antibody to simulate activation of T-cells via the T-cell receptor. Anti-CD3 was co-plated in the presence of IgG control, wild-type mPD-L1 or mPD-L1 mutants and the activation of CSFE-labeled primary CD4^+^ mouse T-cells was measured. In the context of anti-CD3-mediated CD4^+^ T-cell activation, wild type mPD-L1 inhibits activation compared to isotype control (Fig 3D), while mPD-L1 mutants with reduced levels of mPD-1 binding showed a significantly reduced ability to inhibit T-cell activation. In contrast, mPD-L1 mutants with reduced mB7-1 activity elicited effects comparable to wild type mPD-L1. These data suggest that under the *in vitro* experimental system employed, mPD-L1-induced inhibition of CD4^+^ T-cell activation occurs primarily via its interaction with PD-1. Staining isolated CD4^+^ positive T-cells before and after activation with anti-CD3 showed increases in both PD-1 and B7-1 cell-surface expression during the course of activation, consistent with previous reports (S12 Fig)[13]. These data demonstrate the feasibility of generating mutants with specific biological activities that can aid in defining the distinct contributions of the PD-L1:PD-1 and PD-L1:B7-1 interactions to mammalian immunity.

### mPD-L1 binds at the dimer interface of mB7-1

**Fig 4:**
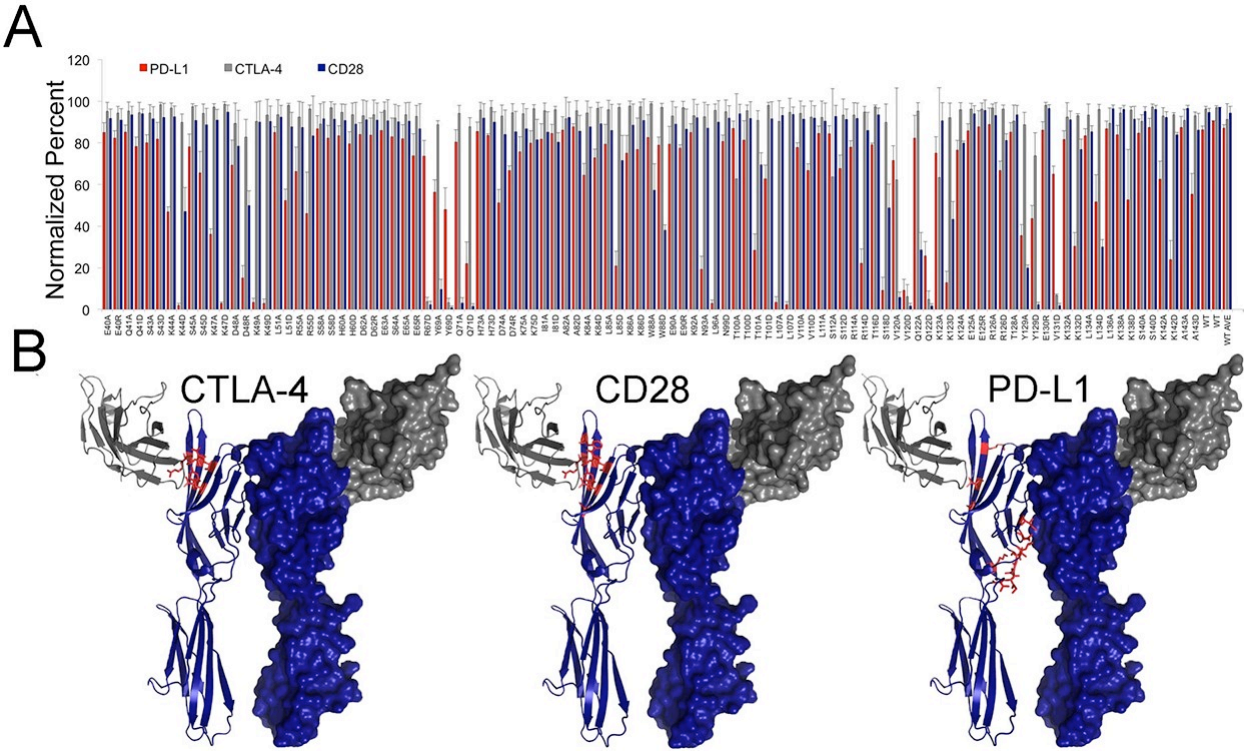
Screening mB7-1 mutants for binding to mCTLA-4, mCD28 and mPD-L1. A) HEK 293 Freestyle cells were transiently transfected with wild type or mutant mB7-1 mCherry constructs. 48 hours post transfection cells were diluted to 1x10^6 cells/mL and 0.5 μg of recombinant mCTLA-4, mCD28 and mPD-L1 fc-fusion protein were added to 100, 000 cells in 100uL in 96-well V-bottom plates. After binding for 1 hour at room temperature, mixing at 900rpm, cells were washed 2X with 1X PBS and 0.2% BSA and subsequently incubated with 0.25 μg of anti-mouse Alexa 488 (Invitrogen) for 30 min. After secondary antibody incubation cells were washed two more times with 1X PBS and 0.2% BSA and analyzed by flow cytometry to quantify the percent of mCherry positive cells (expression) positive for Alexa 488 staining (binding). Data shows three independent experiments with standard deviations. S3 Table highlights mutants in red showing <20% binding and in yellow showing <50 and >20% binding. B) Equivalent residues identified as critical for binding (<20% bound) CTLA-4, CD28 and PD-L1 were highlighted as red on the crystal structure of the hB7-1:hCTLA-4 (PDB: 1I8L).

We utilized a strategy analogous to that described above to map mPD-L1 ligand binding residues to identify mB7-1 residues critical for binding to mCTLA-4, mCD28 and mPD-L1. However, instead of utilizing decorated microbeads, in these mapping experiments we directly assayed binding of Fc-fusion proteins to HEK cells expressing the mB7-1 mutants and detected binding using a fluorescent secondary antibody. Using the crystal structures of hB7-1 (PDB: 1DR9) and the murine B7-1 IgV domain (4RWH), we identified solvent accessible residues on mB7-1 (GetArea algorithm, cutoff ratio 30%). From this list, we generated a set of 152 mB7-1 mutants covering both the IgV and IgC domains. Expression validation in HEK 293 cells confirmed that greater than 95% of the mB7-1 mutants showed expression levels and localization comparable to wild type (S13 Fig). This panel of mutants was screened for binding to recombinant mCTLA-4 mIgG2a, mCD28 mIGG2a and mPD-L1 mIgG2a by flow cytometry (Fig 4A, S14 Fig and S3 Table). From the mCTLA-4 screen, we identified R67, Y69, V120, Q122 and V131 as critically important residues on mB7-1 for binding CTLA-4 (≤20% wild type binding). Mapping these residues onto the hB7-1:hCTLA-4 complex crystal structure (PDB: 1I8L) shows they all reside at the observed binding site for CTLA-4 (Fig 4B). Similarly, screening the mB7-1 mutants with mCD28 identified the same residues as those observed in the mCTLA-4 screen, as well as additional residues, Q71, W88 and Y129. These results support a competitive binding model in which CD28 binds to the same site on mB7-1 as CTLA-4, and also suggests that CD28 might make additional contacts or adopt a slightly different pose than CTLA-4 when bound to mB7-1. Screening with soluble mPD-L1 unexpectedly identified residues residing on the mB7-1 dimerization surface as being critical for binding (K44, K47, D48, K49, N93, L96, L107, S198). Furthermore, most of the mutants we identified as important for CTLA-4 and CD28 (R67D, Y69D, Q122A/D, V131D) were not critical for mPD-L1 binding with the exception of S118D, V120D and K1232D, which did show significant loss of mPD-L1 binding. These mapping data suggest that mPD-L1 and mCTLA-4/mCD28 bind on opposite sides of mB7-1.

### mPD-L1 does not compete with mCTLA-4 or mCD28 for binding to mB7-1 on beads

**Fig 5:**
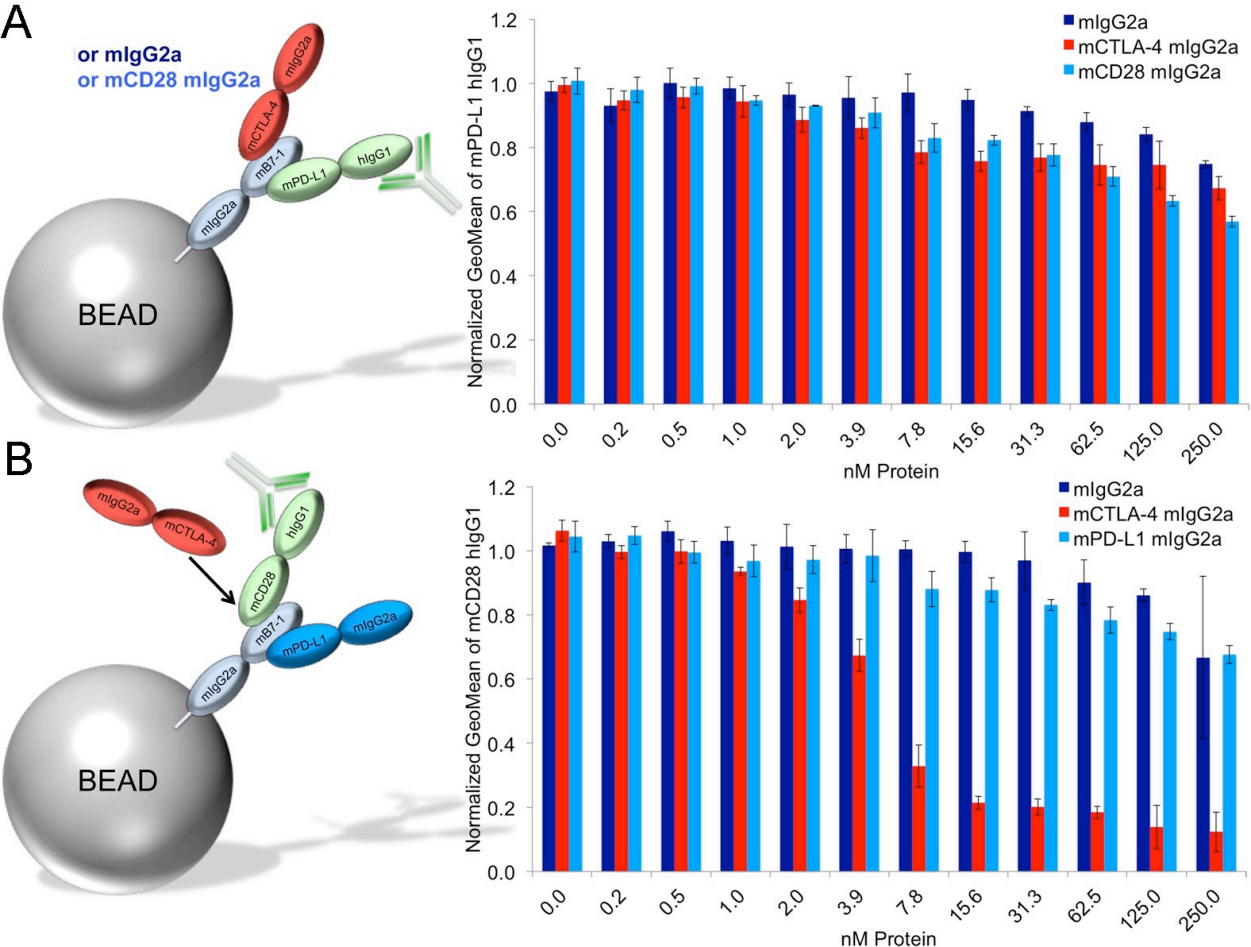
PD-L1 does not compete with CTLA-4 or CD28 for binding to B7-1 on beads. A) Cartoon depiction of the competition assay. Briefly, protein A beads were saturated with mB7-1 mIgG2a protein and subsequently incubated with 20nM mPD-L1 hIgG1 and either an increasing concentration of either mIgG2a, mCD28 mIgG2a or mCTLA-4 mIgG2a. Binding of mPD-L1 was monitored using an anti-human Alexa 488 antibody. B) Same as in A except that 20nM mCD28 hIgG1 was added to coated beads in the presence of increasing concentrations of mIgG2a, mCTLA-4 mIgG2a or mPD-L1 mIgG2a.

Our mapping results show mPD-L1 binding to the homodimer interface of mB7-1, opposite the binding sites for mCTLA-4 and mCD28. Given this binding arrangement, it would be expected that mCTLA-4 and mCD28 do not directly compete with mPD-L1 for binding to mB7-1. This prediction was tested using the bead competition-binding assay and indeed in the presence of saturating mPD-L1 hIgG1, neither mCTLA-4 nor mCD28 competed with PD-L1 for binding to mB7-1 decorated beads (Fig 5A, S15 Fig). In contrast, using saturating mCD28 hIgG1, mCTLA-4 directly competed with mCD28 for binding to the mB7-1 loaded beads. However, mPD-L1 did not exhibit substantial competition with mCD28 or mCTLA-4 for binding (Fig 5B, S15 Fig).

### mPD-L1 and mB7-1 interact in cis

**Fig 6:**
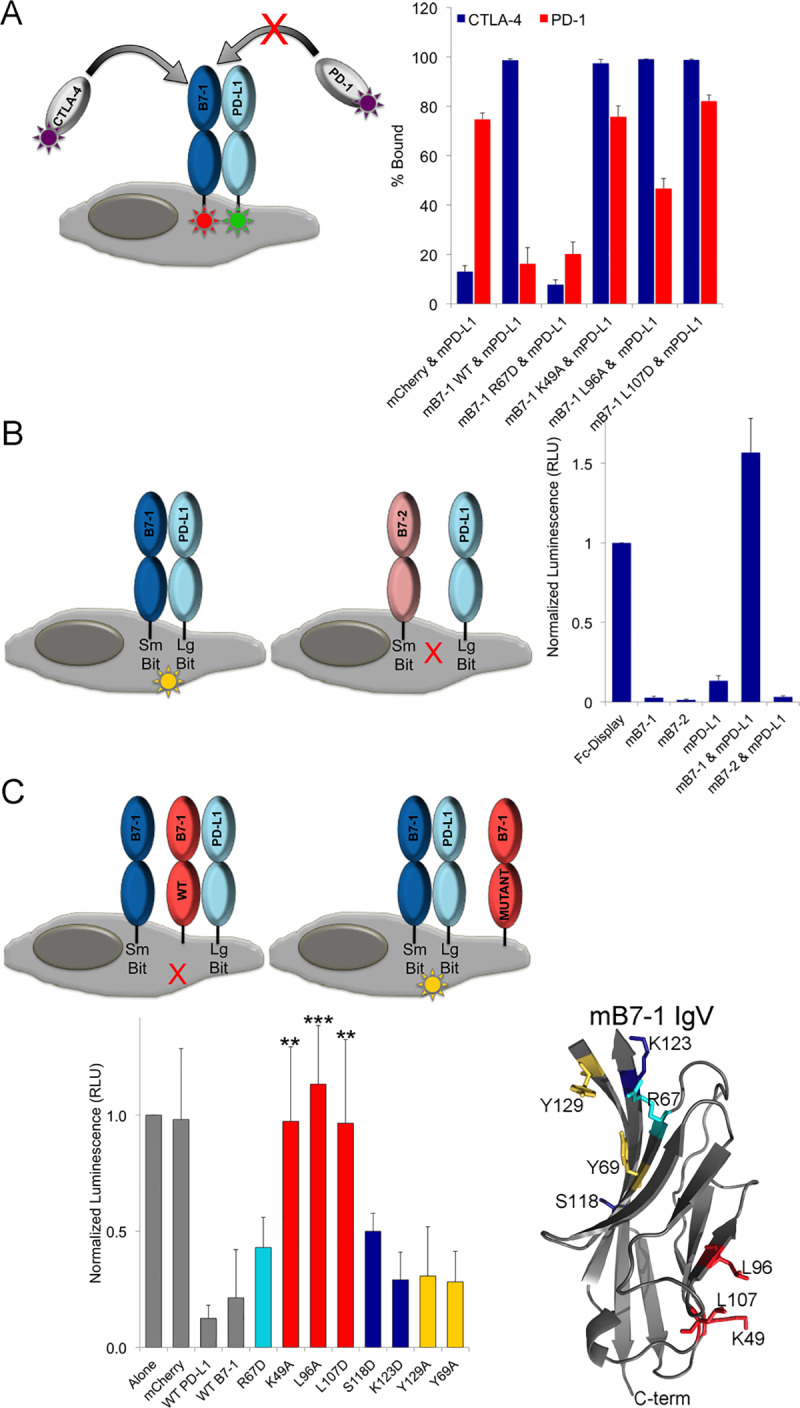
PD-L1 binds in cis to the dimer interface side of B7-1. A) HEK 293 freestyle cells were transiently co-transfected with mB7-1 mCherry and mPD-L1 GFP constructs as depicted. 48 hours post transfection cells were incubated with mCTLA-4 hIgG1 or mPD-1 hIgG1 protein for 1 hour at room temperature with shaking and subsequently incubated with anti-human Alexa 647 secondary. Binding was determined by flow cytometry. Percent bound was calculated as the percent of all double mCherry and GFP positive cells also positive for Alexa 647 staining. Data represents three independent experiments with standard deviations. B) HEK 293 freestyle cells were co-transfected with SmBit and LgBit constructs as indicated. Two days post transfection cells were counted and 50,000 cells were incubated with 12.5 μL of diluted live glo nanoluciferase substrate (Promega) and incubated for 5 min. After incubation luminescence was detected on a Perkin Elmer Envision plate reader. Data was normalized to the luminescence observed for the displayed Fc-dimer constructs and represents 8 independent experiments and standard deviation. C) Same as in B) except that in addition to SmBit B7-1 and LgBit PD-L1, cells were also transfected with the additional constructs depicted. Data was normalized to the luminescence observed for cells expressing mB7-1 SmBit and mPD-L1 LgBit alone and represents 8 independent experiments with standard deviation.

Next we examined whether mB7-1 and mPD-L1 could bind in cis using a co-transfection competition experiment. The hypothesis was that if mB7-1 and mPD-L1 bind in cis then binding of mPD-1 protein would be reduced (compared to no mB7-1 present), as our competition data demonstrate that mPD-1 directly competes with mB7-1 for binding to mPD-L1. We further hypothesized that mCTLA-4 binding would not be affected as our competition data indicated its binding was mPD-L1-independent in the bead-based assay. For this assay, HEK cells were transiently transfected to co-express mB7-1 mCherry (or mutant) and mPD-L1 GFP, and were subsequently queried for binding to mCTLA-4 hIgG1 or mPD-1 hIgG1 protein (Fig 6A). Our results for cells co-expressing wild-type mB7-1 and mPD-L1 showed significantly reduced mPD-1 binding compared to cells transiently co-expressing mCherry and mPD-L1 (control). In contrast, mCTLA-4 binding to cells co-expressing mB7-1 and mPD-L1 was unaffected. In cells co-transfected with mPD-L1 and mB7-1 R67D, reduced mPD-1 binding was still observed because this mB7-1 mutant retains wild type mPD-L1 binding activity, but we observed a loss of mCTLA-4 binding as this mutant showed <20% binding to mCTLA-4 in the mutant screen. In contrast, co-expression of mPD-L1 with mB7-1 mutants K49A, L96A or L107D, all of which reside at the mB7-1 dimer interface, resulted in a significant rescue of mPD-1 binding as all three of these mB7-1 mutants showed <20% binding to mPD-L1 in the bead-based screen, while maintaining wild-type mCTLA-4 binding. Importantly, we have been unable to demonstrate a trans interaction between mPD-L1 and mB7-1 (S16 and S17 Figs), eliminating this trans interaction as the mechanism for impaired mPD-1 binding. These data confirmed that mPD-L1 and mB7-1 interact in cis and that residues contributing to the mB7-1 dimer interface side are involved in the recognition interface responsible for the PD-L1:B7-1 cis interaction.

Freeman and colleagues recently reported the use of a split nanoluciferase assay (Promega) to demonstrate that hPD-L1 and hB7-1 could participate in a cis interaction on the same plasma membrane [17]. To confirm that mouse B7-1 and PD-L1 also interact in cis and to further support our results from co-transfection experiments, we implemented this strategy. This approach is appealing because it utilizes vectors driving expression from the weak HSV TK mammalian promoter, resulting in lower levels of protein expression, which more closely recapitulate endogenous levels. Additionally, the assembly of SmBit and LgBit nanoluciferase fragments is completely reversible in real time, which allowed us to examine potential effectors of the cis mPD-L1:mB7-1 complex. We designed split nanoluciferase SmBit and LgBit constructs for full-length mB7-1, mB7-2, and mPD-L1, mCTLA-4 and mPD-1, as well as a positive control containing the Fc domain of mIgG2a Fc fused to the transmembrane of mPD-L1 (Fc-Control). Co-transfection of SmBit and LgBit Fc-control, which results in expression of cell surface displayed covalent dimer, efficiently drove assembly of the nanoluciferase fragments and yielded very high luminescence (Fig 6B). In comparison, co-transfection of SmBit and LgBit constructs of each of mB7-1, mB7-2 and mPD-L1 all resulted in low luminescence; however, co-transfection of SmBit mB7-1 and LgBit mPD-L1 resulted in very high luminescence comparable to that observed for the Fc-Display control. We also observed very low luminescence in cells co-transfected with SmBit mB7-2 and LgBit mPD-L1.

To evaluate whether the observed luminescence was specifically due to cis binding between mPD-L1 and mB7-1, we co-transfected in a third construct along with the SmBit mB7-1 and LgBit mPD-L1 (Fig 6C). Additional co-transfecting with either WT mPD-L1 or WT mB7-1 significantly reduced the luminescence indicating effective “cis” competition for binding SmBit mB7-1 or LgBit mPD-L1, respectively. Co-transfection with the mB7-1 R67D mutant showed effective competition, as this mutant retains mPD-L1 binding capability (Fig 6C, light blue bar). In contrast, co-transfection with the mB7-1 K49A, L96A and L107D mutants showed significant preservation of the luminescence signal compared to wild-type mB7-1, as these mutants exhibit reduced mPD-L1 binding in the screen (Fig 6C, red bars). To rule out the possibility that trans binding between PD-L1 and B7-1 was somehow driving the observed luminescence signal we added PD-L1 mCherry or B7-1 mCherry expressing cells to cells expressing smBit/ LgBit B7-1 or smBit/LgBit PD-L1 or smBit B7-1/LgBit PD-L1. Addition of trans presenting B7-1 or PD-L1 had no effect on the luminescence of B7-1 alone, PD-L1 alone or B7-1/PD-L1 cis complex, suggesting trans binding is not occurring in this system and that neither B7-1 nor PD-L1 can compete in trans with Cis bound B7-1/PD-L1 (S17 Fig).

We also examined mutants of mB7-1 identified in the mapping experiment that showed reduced mPD-L1 binding but are located in the CTLA-4/CD28 binding site not at the dimer interface of mB7-1. In our mB7-1 mapping experiments using soluble mPD-L1 Fc-fusion protein, mB7-1 mutants, S118D and K123D, showed less than 20% mPD-L1 binding (S3 Table), while the Y129A and Y69A mutants showed more modest loss of PD-L1 binding (S3 Table). However, in the context of the cis mPD-L1:mB7-1 complex, none of these mutants significantly rescued luminescence and instead showed a signal comparable to co-transfection with wild-type mB7-1 (S118D and K123D –Blue Bars and Y129A and Y69A –Yellow Bars, Fig 6C), suggesting these residues are not critical for binding mPD-L1 in cis, in the context of the plasma membrane. These observations support our findings that residues located on the homodimerization surface of mB7-1 are predominantly involved in forming contacts with PD-L1 in cis in a cellular context.

### mCTLA-4 and mCD28 alter the organization of mB7-1 and mB7-2 on the cell surface

**Fig 7:**
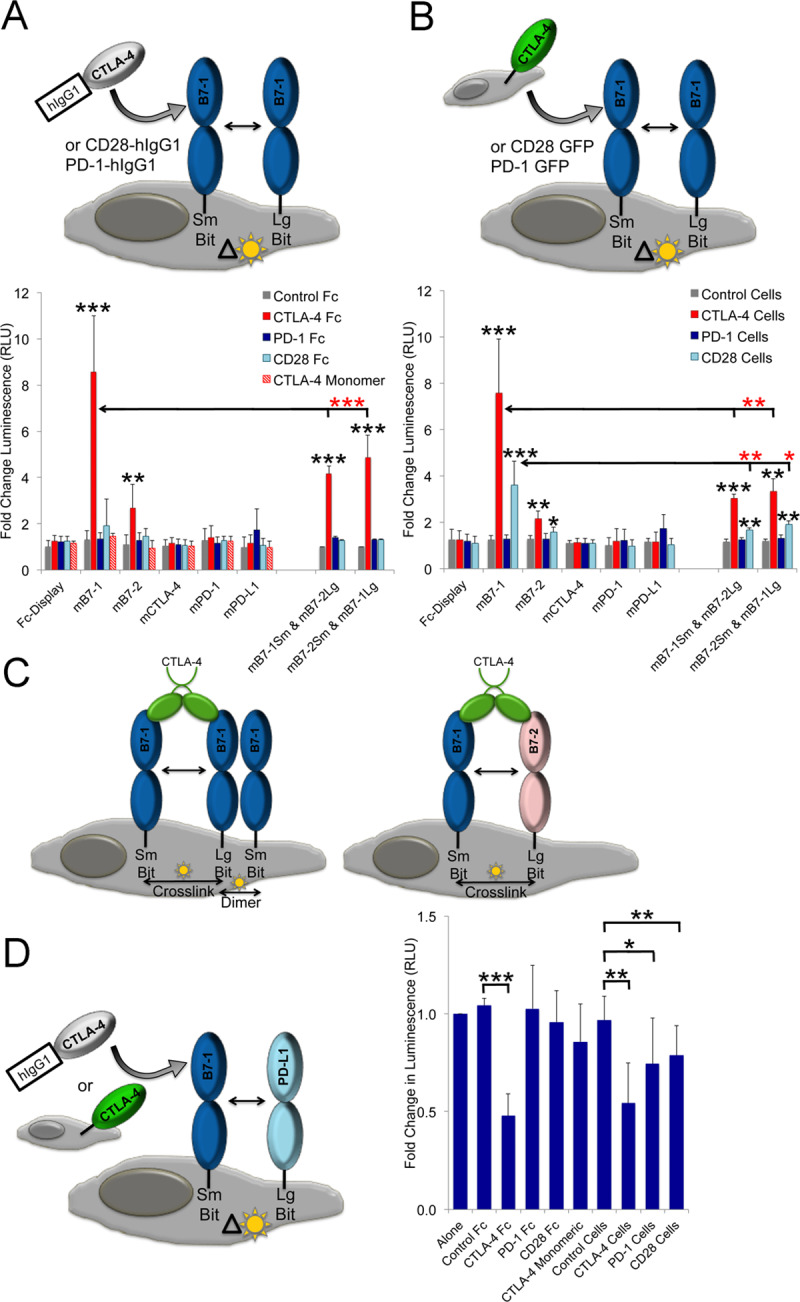
Reorganization of cell surface B7-1 inhibits the cis interaction between PD-L1 and B7-1. A) HEK 293 freestyle cells were co-transfected with SmBit and LgBit constructs as indicated. Two days post transfection 100,000 cells were incubated with 0.5 μg of recombinant CTLA, CD28 or PD-1 or control Fc-fusion protein in 150 μL reaction volume. After binding for 1 hour at room temperature a 50 μL aliquot from each binding reaction was removed and 12.5 μL of nanoluciferase substrate added. Data shows the fold change calculated as the ratio of stated condition to untreated cells and represents 8 independent experiments with standard deviations. B) Same as in A) except that 50,000 HEK 293 cells expressing either CTLA-4 GFP, CD28 GFP or PD-1 GFP or control were added as indicated. C) Same as in A) and B) except querying HEK 293 cells transiently transfected with B7-1 SmBit and PD-L1 LgBit constructs. Significance calculated by one-way Annova is indicated by stars, *** = P <0.001, ** = P<0.01 and * = P<0.05.

Using the split nanoluciferase system, we examined the effect of mCTLA-4, mCD28 and mPD-1 receptor binding on the organization and behavior of ligands on the cell surface. Adding either soluble recombinant mCTLA-4 hIgG1 protein (Fig 7A) or mCTLA-4 GFP-expressing cells (Fig 7B) to cells co-expressing SmBit and LgBit mB7-1 or SmBit and LgBit mB7-2 resulted in significant increases in luminescence compared to controls (CTLA-4 hIgG1; P_B7-1_<0.001, P_B7-2_ <0.01, CTLA-4 Cells; P_B7-1_<0.001, P_B7-2_<0.01). However, the fold change for mB7-1 was ~3 times higher than that of mB7-2. Soluble monomeric CTLA-4 was not capable of inducing increases in luminescence signals, demonstrating the requirement for a dimeric effector. Addition of soluble mCD28 hIgG1 did not cause any enhancement in luminescent signal; however, addition of mCD28 GFP expressing cells did cause a significant increase in luminescence for both mB7-1 and mB7-2 (P_B7-1_<0.001, P_B7-2_<0.05), but these increases were 2.3-fold less than that observed with mCTLA-4 expressing cells. Addition of either mPD-1 hIgG1 protein or mPD-1 GFP-expressing cells did not have a significant impact on the luminescence of any of the SmBit/LgBit pairs examined in Fig 7A and 7B.

To examine the relative contribution of “crosslinking” and homodimer formation to the increase in luminescence observed for mB7-1 and mB7-2 in the presence of CTLA-4 and CD28, we co-transfected SmBit mB7-1 with LgBit mB7-2 and vice versa (Fig 7A–7C). As mB7-1 and mB7-2 do not dimerize with each other, any signal observed upon the addition of CTLA-4 and CD28 must only result from crosslinking. Interestingly, a significant increase in luminescence was observed upon adding CTLA-4 either as soluble protein or on cells, but only for CD28 when it was cell surface expressed. However, the increase in B7-1/B7-2 crosslinked luminescence signal was 2–3 times higher for CTLA-4 than CD28 supporting previous proposals that CTLA-4 is more effective at crosslinking the B7-1/B7-2 ligands [33]. Furthermore, the changes in luminescence observed when CTLA-4 and CD28 expressing cells were added to the B7-1/B7-2 co-expressing cells were significantly lower than those observed when mB7-1 SmBit and mB7-1 LgBit were co-expressed (Red stars; P_CTLA-4_<0.01 (for both comparisons), P_CD28_<0.01,and 0.05 for comparison to cells expressing B7-1 SmBit/B7-2 LgBit and B7-1 LgBit /B7-2 SmBit respectively), suggesting the possibility that in addition to crosslinking (or minimal crosslinking in the case of CD28), both receptors may be capable of enhancing mB7-1 dimer formation (i.e., enhancement in luminescence above that observed from crosslinking alone).

### mCTLA-4 and mCD28 engagement with mB7-1 reduces cis PD-L1 binding

In a similar set of experiments, we examined the effect of receptor binding on the cis mPD-L1:mB7-1 interaction. Unexpectedly, we observed a significant reduction in the mB7-1 SmBit:mPD-L1 LgBit luminescence in the presence of either soluble CTLA-4 hIgG1 protein or CTLA-4 GFP-expressing cells (P_protein_<0.001, P_cells_ <0.01) (Fig 7D). No significant effect was observed upon addition of mCD28 hIgG1 or mPD-1 hIgG1. A more modest, but still significant, reduction was observed upon adding mPD-1 GFP (P<0.05) and mCD28 GFP (P<0.01) expressing cells.

## Conclusion

Binding promiscuity is increasingly appreciated as a major mechanistic feature of the processes underlying multi-cellularity and is particularly important to a range of innate and adaptive immune responses. We implemented high-throughput cell microarray- and FACS-based platforms to examine the promiscuous binding interactions of mPD-L1 with mPD-1 and mB7-1, define the respective protein recognition interfaces on mPD-L1 and mB7-1, and to generate selective mPD-L1 and mB7-1 mutants with distinct biochemical and functional properties. The cell microarray platform provided confirmation that both mPD-1 and mB7-1 are ligands for mPD-L1 and identified a series of mPD-L1 point mutants that are highly selective for binding only mB7-1 (D122A, Y123A, Y123R, K124A/D/R, R125A/D), only mPD-1 (D49R, V54D/R, Y56A/D/R, E72R, G119D, G120D), or which are defective in binding both mB7-1 and mPD-1 (T37A/R, L53D/R, I115D, I116R, A121D/R, D122R, Y123D). While the cell microarray platform benefits from high-density and rapid analysis, it is limited with respect to its dynamic range. As the panel of mPD-L1 mutants was expected to display a wide range of affinities, we adopted a high-throughput FACS approach in which the mPD-L1 mutants were expressed and presented on cells, and queried with multivalent microbeads decorated with either mPD-1 or mB7-1. mPD-L1 mutants evaluated in this fashion exhibited behavior consistent with the initial microarray experiments and afforded a more faithful assessment of the relative binding propensities. We also examined mPD-L1 variants harboring mutations in the IgC domain and identified only one mutant with a strong effect on binding (T185D). Although it is possible mB7-1 forms a direct contact with this IgC residue, it is more likely this mutant impacts overall stability or orientation of the IgC or IgV domain, which impacts mB7-1 binding.

For a subset of the mPD-L1 mutants, purified Ig-fusion proteins were used in FACS-monitored titrations to estimate binding relative to the wild type protein. As illustrated in Fig 3, mPD-L1 variants exhibited a range of EC50s for mPD-1 and mB7-1; for example, two mutants (K124A and R125A) exhibited an ~20-fold reduction in binding to PD-1 while these same mutants showed either near wild-type or 2-fold enhanced binding to mB7-1. More interesting are the mutants that exhibit extreme selectivity, such the PD-L1 mutants D49R, Y56A, G119D and G120D, which showed wild type binding to PD-1, but no detectable binding to mB7-1 over the concentration range examined. Analogously, the mPD-L1 mutants D122A and Y123R showed wild type binding to mB7-1, but no detectable binding to mPD-1. The dissection of biochemical function afforded by these mutants provides new tools to assess the biological contributions of the PD-1- and B7-1-associated signaling processes.

In a conventional αCD3-driven CD4^+^ T-cell activation assay, those mPD-L1 mutants unable to bind mPD-1 could no longer support inhibition of T-cell activation, while those defective in mB7-1 binding did support inhibition comparable to wild type mPD-L1. These data indicate that, in the context of this particular assay format, engagement of PD-1 is the predominant PD-L1-driven inhibitory pathway, suggesting that the activities of the PD-1 and B7-1 specific PD-L1 pathways are distinct. Robust cell-surface expression of PD-L1 as well as B7-1 and PD-1 has been observed on mouse CD4+ T-cells after activation[13]. Therefore, it is possible that B7-1 on the T-cell is bound to PD-L1 in cis; however, the impact of this potential interaction on activation in the context of our assay is unknown. Previously reported *in vivo* studies utilizing an αPD-L1 monoclonal antibody that specifically blocks B7-1 binding, but not PD-1 binding, demonstrated a significant role for the PD-L1:B7-1 complex in protecting against transplant rejection and developing sustained immune tolerance [9,12]. However, there is also a wealth of evidence implicating the PD-L1:PD-1 pathway in the promotion of T_reg_ maturation and inhibition of self-reactive T-cells suggesting specific roles for this complex in autoimmunity, as well as in immune evasion in many cancers [34,35]. Thus the relative importance of PD-L1:B7-1 and PD-L1:PD-1 engagement and the interplay between these two distinct activities is likely to be highly context dependent.

Using two different methods we complemented recent work of Freeman and colleagues showing that hPD-L1 and hB7-1 interact in cis on the same cell surface [17]. These results may impact the interpretation of *in vitro* T-cell activation assays where B7-1 or PD-L1 are co-plated with activating antibodies. The development of novel assays designed to look specifically at the impact of cis-interacting PD-L1 and B7-1 will be important for elucidating the functional consequences of this interaction. It is our hope that the mPD-L1 and mB7-1 selectivity mutants generated herein will contribute to this effort. More generally, the demonstration of similar interactions involving the human and murine orthologs suggests the PD-L1:B7-1 cis interaction is conserved among mammals.

The PD-L1 ectodomain is composed of consecutive N-terminal membrane distal IgV and membrane proximal IgC domains. The IgV domain, which is primarily responsible for binding PD-1 and B7-1, exhibits typical IgV domain topology, with front β-sheet (strands GFCC’C”) and back β-sheet (strands ABED) forming a two layered β-sandwich. The PD-L1:PD-1 crystal structures demonstrate that the binding interface is formed predominately by residues contributed from the front faces (i.e., GFCC′β-strands) of PD-1 and PD-L1 [32,36], and show that the PD-L1 mutations associated with reduced binding to PD-1 (D28, D122, Y123, K124 and R125) were buried at the crystallographically observed binding interface. Although we identified several residues involved in binding both PD-1 and B7-1 (T37, L53, I115, I116, G120, A121), we also identified several (D49, V54, Y56, Q66, E72, G119) that only impacted B7-1 binding, suggesting that while the PD-1 and B7-1 binding sites overlap, they are distinct. This model is consistent with competition experiments in which PD-1 and B7-1 competed for binding to PD-L1.

Murine B7-1 IgV is similar in overall organization to PD-L1, composed of front and back β-sheets consisting of AGFCC′C′′ and DEB and β-strands, respectively. Defining the residues and surfaces contributing to the PD-L1:B7-1 binding interface is particularly important due to the current lack of a crystal structure for the PD-L1:B7-1 complex. Using a strategy similar to that for mPD-L1, we generated a panel of mB7-1 mutants and queried binding to mCTLA-4, mCD28 and mPD-L1. We correctly identified residues R67, Y69, V120, Q122 and V131 on mB7-1 as critically important for mCTLA-4 binding; all of these residues are directly involved in binding CTLA-4 as observed in the crystal structure of the CTLA-4:B7-1 complex [37]. These same residues were identified as important contributors to binding mCD28. Interestingly, we also identified Q71 and Y129 as residues important for CD28 binding, but not CTLA-4. It is well established that CTLA-4 and CD28 compete for binding with similar modes of B7 recognition, consistent with our *in vitro* competition data. However, our detailed mapping suggests there are modest differences in the contacting residues in the CTLA-4 complex versus the CD28 complex, as two mB7-1 mutants (Q71A and V120A) only exhibited lost binding to CD28. Most interesting were the results obtained from querying the mB7-1 mutants with mPD-L1, in which a very different set of residues were identified as being important for binding (K44, K47, D48, K49, N93, L96, L107, S118, V120, K123 and S198). Mapping these residues to the crystal structure of the CTLA-4:B7-1 complex shows the majority of these residues lie on the surface contributing to the B7-1 homodimer interface near the hinge region between the IgV and IgC domains. We also identified a small number of residues (S118, V120 and K123) located near the CTLA-4/CD28 binding interface; however, experiments examining the cis B7-1 and PD-L1 interaction (Fig 6) shows that these residues are likely not important for the formation of the cis complex in a cellular context and support a model in which PD-L1 is binding a surface that overlaps with the dimer interface of B7-1.

Our mB7-1 mapping data suggested mPD-L1 would not directly compete with mCTLA-4 or mCD28 for binding to mB7-1, which was consistent with our *in vitro* bead-based competition experiments, as well as previously reported competition SPR data [38]. Interestingly, using the split nanoluciferase system we were able to assess changes in the organization of mB7-1 and mB7-2 on the cell surface resulting from interaction with mCTLA-4 and mCD28. It has long been thought that CTLA-4 induces B7 crosslinking, in which each subunit of the CTLA-4 dimer can interact with a B7 ligand forming a bridge between the two B7 molecules [39,40]. Unlike B7-2, B7-1 can also dimerize with a relatively weak affinity of ~20–50 μM [41], and crosslinking by homodimeric bivalent CTLA-4 has been proposed to generate bridged dimers with each CTLA-4 monomer binding to one B7-1 monomer from two distinct B7-1 dimers. CD28 is also homodimeric, however, structure-based comparisons to the CTLA-4 homodimer have suggested that the extended dimer interface of CD28 may place steric limitations that prevent CD28 from crosslinking two B7 ligands (i.e., the CD28 dimer is monovalent for the B7 ligands) [33]. In contrast, recent molecular modeling of the CD28 monomer as well as experiments looking at CD28 avidity upon TCR engagement suggest that crosslinking by CD28 may be energetically possible and context dependent [42,43].

Our results show dimeric CTLA-4 (soluble Ig-fusion protein or cell surface expressed) reorganizes B7-1 and B7-2 on the cell surface. The increase in luminescence observed was due, at least in part, to crosslinking because soluble monomeric CTLA-4 was unable to exhibit the same effect for either ligand. Interestingly, we observed an ~3-fold higher increase in luminescence for B7-1 (dimeric) compared to B7-2 (monomeric) suggesting that the valency of the ligand also contributes to or effects the luminescence signal. To further explore the relative contributions of ligand valency and crosslinking, we compared the effect of each receptor on cells expressing only B7-1 (SmBit + LgBit) or cells expressing B7-1 and B7-2 (SmBit + LgBit). As B7-1 and B7-2 do not heterodimerize, the increase in luminescence observed must be due solely to reconstitution of the SmBit and LgBit fragments between crosslinked B7 molecules. The luminescence observed was much higher for CTLA-4 than for CD28, providing further support (though not proof) for the hypothesis that although CD28 is dimeric it binds monovalently to B7 ligands and does not efficiently crosslink B7 ligands [33,44]. In addition, the signal we observed upon addition of soluble CTLA-4 or CD28 to cells expressing B7-1 and B7-2 (crosslink dependent signal) was ~3-fold lower than that observed when these soluble proteins were addred to cells expressing only B7-1 (SmBit + LgBit). One interpretation of this observation is that receptor engagement of B7-1 directly effects valency and that the higher signal observed for B7-1 alone is the result of additional SmBit and LgBit association within individual B7-1 dimers (intradimer). It is possible that receptor engagement orients or stabilizes the extracellular domain in a way that increases formation of the relatively low affinity B7-1 dimer [41]. However, in an indirect assay such as the one employed here, we cannot rule out other possible mechanisms for the observed enhancement in luminescence (i.e., other conformational changes or orientational restrictions that bias SmBit and LgBit reconstitution). Therefore, additional work will be needed to explore the potential impact of CTLA-4 and CD28 binding on B7-1 dimerization. However, it is clear from our data that while both CTLA-4 and CD28 reorganize B7-1 and B7-2 on the cell surface they do so in very distinct ways.

Most surprising was the dramatic effect mCTLA-4 binding had on the cis interaction between mB7-1 and mPD-L1. Although our data and previously reported data [38] clearly demonstrate that PD-L1 and CTLA-4 or CD28 do not directly compete for binding, the proposed reorganization of mB7-1 on the cell surface by mCTLA-4 (and to a lesser extent mCD28) results in a decrease in mPD-L1 binding to mB7-1 in cis. As our mapping data supports a model in which mPD-L1 binds to the mB7-1 dimer interface it is possible that formation of mB7-1 dimer would sterically occlude and compete with mPD-L1 binding. Our competition data shows that ternary murine CTLA-4/B7-1/PD-L1 and murine CD28/B7-1/PD-L1 complexes can be formed using recombinant protein *in vitro*, yet we do not know whether these complexes can form in the context of cell-cell contacts associated with engagement of T-cells and APC cells, how stable they might be or how effectively PD-L1 cis binding is reduced. Our experiments do show that addition of mCTLA-4 results in stronger dissociation of the murine PD-L1:B7-1 cis complex than mCD28, which may reflect the differences we observed in the effect each receptor had on the cell surface organization of mB7-1, perhaps as the consequence of their different valencies. Also notable is the inability of soluble mPD-1 and relatively weak ability of cell-surface expressed mPD-1 to compete with mB7-1 for cis bound mPD-L1. This result was surprising given the reported affinity for PD-1 binding to PD-L1 is ~2 times higher than that of B7-1 [8] and suggests the effective affinity of the cell-based cis complex is likely higher than that observed with soluble proteins.

**Fig 8:**
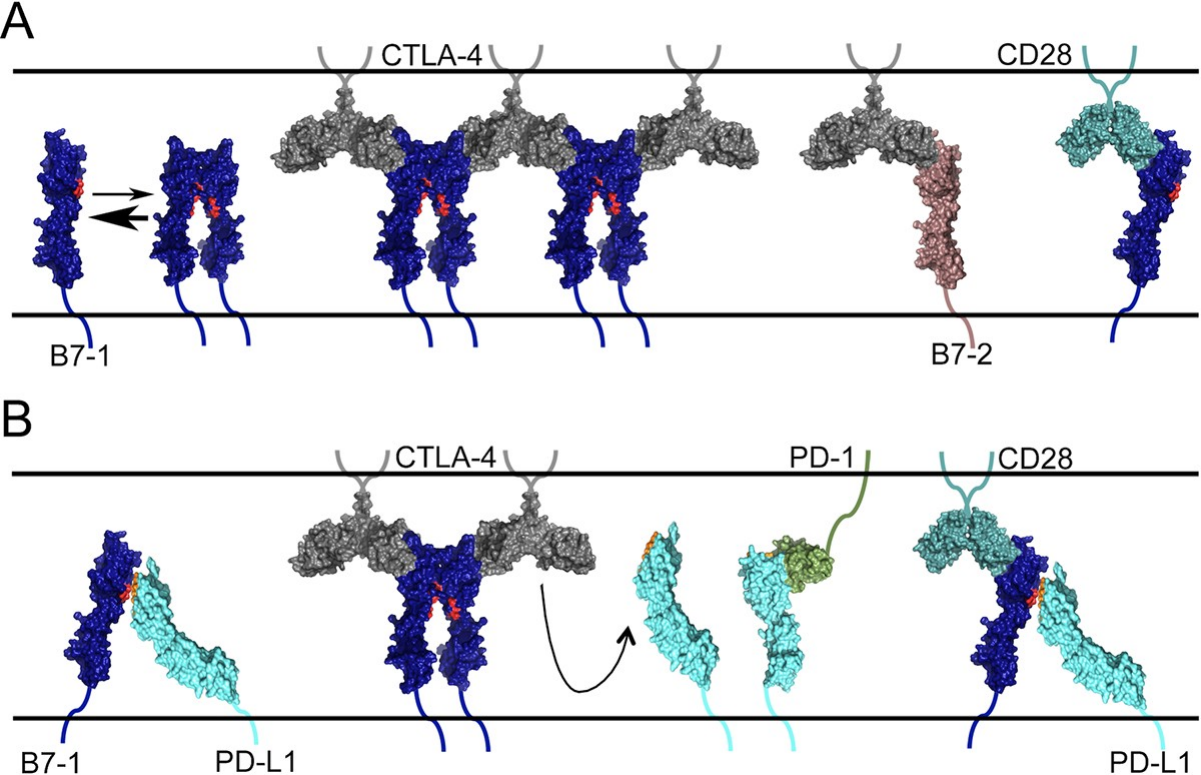
Proposed model for the regulation of the cis bound PD-L1:B7-1 complex. A) B7-1 exists in a monomer-dimer equilibrium. Binding of CTLA-4 cross-links cell surface B7-1 thereby promoting and/or stabilizing B7-1 dimer formation. B7-2 does not dimerize and CD28 may not cross-link B7-1 to the same extent as CTLA-4. B) B7-1 and PD-L1 interact in cis and engagement of CTLA-4 with B7-1 inhibits PD-L1 binding, freeing it to bind PD-1. A tertiary complex may form between CD28, B7-1 and PD-L1 but has not been observed directly. The crystal structures used in the generation of this model cartoon include: 1DR9, 1I8L, 4R0l, 1I85, 4Z18 and 3SBW.

Together our results suggest a model in which engagement of mB7-1 by mCTLA-4, and to a lesser extent mCD28, results in a reorganization of cell surface expressed mB7-1 in a fashion that negatively impacts the ability of mPD-L1 to bind to mB7-1 in cis (Fig 8). To further probe this model additional studies are required to 1) assess the potential for cell-cell ternary complex formation; 2) examine the effect of receptor binding on the valency and organization of B7-1 and B7-2 on the cell surface and 3) determine the impact of these interactions on T-cell function. Also, the inability of mPD-1 to compete with mB7-1 for cis bound mPD-L1 in both our co-transfection competition binding experiment and in the split-luciferase experiment suggests that mB7-1 might act as a molecular trap for mPD-L1, biasing formation of a bound state that makes it less accessible for mPD-1 binding, and that engagement by mCTLA-4 and mCD28 may differentially regulate the accessibility of mPD-L1 to mPD-1. Therefore, the cis bound complex could act as a tuning mechanism for balancing the inhibitory and stimulatory responses and might have significant implications for immune regulation by CTLA-4 and CD28. Supporting this hypothesis is data showing overexpression of B7-1 in PD-L1-expressing melanoma tumor cells prevented PD-L1 from binding to PD-1 and reduced the ability of these tumor cells to suppress T-cell activation [19,20,45]. In this specific experimental context, it would be interesting to determine whether addition of soluble CTLA-4 or CD28 reverses this effect. Additionally, recent work has demonstrated the ability of the cis PD-L1:B7-1 complex to restrict PD-1 activity in both autoimmune EAE and anti-tumor immunity models [18]. Our work suggests CTLA-4 may drive the release of cis bound PD-L1 providing a mechanism by which PD-1 signaling may be modulated.

Our model also has implications for the mechanistic interpretation of CTLA-4 based therapeutics. Abatacept (Bristol-Myers Squibb, Orencia ®) and Belatacept (Bristol-Myers Squibb, NULOJIX®) are two FDA approved biologics, consisting of the extracellular domain of CTLA-4 fused to the Fc domain from human Ig, used to treat autoimmune diseases and kidney transplant rejection, respectively [46–48]. These therapeutics are thought to dampen the immune response by binding and sequestering B7-1 and B7-2 ligands, and thereby preventing their engagement with the CD28 costimulatory receptor. However, our data shows that CTLA-4 Fc-fusion protein also reorganizes cell surface B7-1 and B7-2 and reduces cis PD-L1 and B7-1 complex formation. Therefore, it is possible that by engaging B7-1, these therapeutics also release cis bound PD-L1 making it more accessible for binding to PD-1 on T-cells which would also suppress activation. Understanding the detailed mechanisms by which these therapeutics elicit their effects will be critical for the development of next generation therapies with better efficacy and reduced off target side effects. To this end, the specificity mutants we identified will prove invaluable tools in that effort.

Our approach successfully identified selectivity mutants for mPD-L1 and mB7-1 and is readily applicable to other major immune regulators. The reagents generated provide the foundation for the creation of new murine model systems to examine the *in vivo* roles of these intersecting signaling pathways, and afford the opportunity to develop biologics with modified selectivities and/or affinities, which could translate into more efficacious treatments with new indications and fewer deleterious side effects. By expanding our expression library to include other key cell surface proteins (i.e. GPCRs, growth factor receptors, cytokines) this approach can be used in the future to identify and characterize novel or promiscuous protein interactions.

## Supporting information

S1 Method This file contains a detailed description of additional methods used in this manuscript. (PDF)

S1 Fig Fluorescence microscopy images from representative members of the Ig superfamily expression library. The top panel shows 24 representative images of HEK 293 cells expressing different Ig superfamily targets in our Type I mCherry vector. All images were acquired on a EVOS inverted benchtop florescence microscope. Cytosolic mCherry was transfected as a negative control for membrane localization (Top Left rectangle) and the bottom right most rectangle shows untransfected cells. The bottom panel shows a 10X zoomed images of select constructs depicting the difference observed between cytosolic and membrane localized constructs.
(PDF)

S2 Fig Schematic for generating the library. **A)** We employed ligation independent cloning (LIC) methods for the design of custom vectors in which the SacB killer gene is replaced by the full-length ectodomain of a gene of interest flanked by on the N-terminus by the leader sequence from the human erythropoietin gene and flanked on the C-terminus by the transmembrane domain from mouse PD-L1 followed by mCherry fluorescent protein. **B)** The same LIC sites (and therefore the same PCR products) can be used to clone into a separate vector for the expression of Fc fusion proteins for downstream validation experiments.
(PDF)

S3 Fig PD-L1 mutants express to a similar extent as wild-type PD-L1. **TOP** Graph shows the %mCherry positive HEK 293 cells transfected with wild-type PD-L1, mutant PD-L1 or mCherry empty vector control. Data is the average from three independent transfections with error bars showing the standard deviation. **BOTTOM** One-way ANOVA analysis was performed to determine statistically significant differences between each mutant compared to WT PD-L1. To aid in visualizing the results of this analysis, the graph shows the fold change in average expression for each mutant compared to wild-type PD-L1 (normalized to 1). All of the mutants shown in **BLUE** were not statistically different from wild-type, those in GREEN were significantly different but showed higher expression than WT, those in RED were significantly different and showed ~25% less expression than WT.
(PDF)

S4 Fig Fluorescence microscopy and comparative monoclonal antibody binding to select mPD-L1 and mB7-1 mutants. **TOP** HEK 293 suspension cells were transiently transfected with either wild type or mutant mPD-L1 or mB7-1 as indicated in 24-well suspension plates. Two days post transfection cells were imaged for mCherry expression using an EVOS inverted benchtop florescence microscope. **BOTTOM** HEK 293 suspension cells were transiently transfected with either wild type or mutant mPD-L1 or mB7-1 as indicated. Two days post-transfections, 100,000 cells from each transfection were incubated with 0.5ug of each monoclonal antibody (R&D Systems MAB90783 (anti-mPD-L1) and R&D Systems MAB740 (anti-mB7-1) for 1 hour with shaking at room temperature. Cells were subsequently washed three times with 1X PBS with 0.2% BSA and incubated with secondary antibodies (anti-Rabbit 647 (PD-L1) and anti-Rat 647 (B7-1). Cells were analyzed by flow cytometry and data presented as the GeoMean of 647 (bound).
(PDF)

S5 Fig Representative FACS scatter plots showing PD-L1 mutants with altered binding phenotype. Data shows a representative set of FACS scatter plots obtained from the microbead binding experiment. Microbeads coated with either control, PD-1 or B7-1 Fc-fusion protein were used to challenge cells expressing wild-type PD-L1 or mutants. The E60A mutant did not affect binding of PD-L1 to either PD-1 or B7-1. G119D and G120D lost binding to B7-1 but maintained binding to PD-1. The A121R mutant does not bind either PD-1 or B7-1. The D122A, Y123R and R125A mutants all maintained binding to B7-1 but lost binding to PD-1.
(PDF)

S6 Fig Residues on PD-L1 involved in B7-1 binding remain exposed with PD-1 bound. 360 degree rotation of a space filling representation of the PD-1:PD-L1 crystal structure (PDB: 3SBW). Residues are color coded the same as previously described (Green = PD-1 binding null, Red = B7-1 binding null, Gray = Both null). Most of the PD-1 specific residues are buried at the interface within the complex and therefore not visible. In contrast, many of the B7-1 residues remain exposed in the space fill model demonstrating that these positions are not involved in and do not impact the PD-1 binding interface.
(PDF)

S7 Fig PD-1 and B7-1 binding to PD-L1 IgC mutants. **A)** A panel of 65 PD-L1 IgC mutants were examined for binding to mPD-1 (Blue Bars) and mB7-1 (Red Bars) using the microbead binding assay described in the main text. Gray bars depict the %mCherry expression for each mutant normalized to wild-type. All data represents two independent experiments with error bars showing the standard deviation. **B)** Mapping of the IgC mutants onto the structure of PD-L1 (PDB: 3SBW). In the IgV domain the color coding is the same as the main text, green = PD-1 binding affected, red = B7-1 binding affected, gray = both PD-1 and B7-1 binding affected. For the IgC domain T185D showed the most significant effect on B7-1 binding, highlighted red while the other mutants identified showed more modest effects, highlighted yellow. **C)** Table showing the normalized average binding of PD-1 and B7-1 to these select mutants.
(PDF)

S8 Fig PD-1 competes with B7-1 for binding to PD-L1. **A)** Cartoon depiction of the competition assay. Briefly, protein A beads were saturated with mPD-L1 mIgG2a protein and subsequently incubated with 20nM mB7-1 hIgG1 and an increasing concentration of either mIgG2a, mPD-1 mIgG2a. Binding of mB7-1 hIgG1 was determined using an anti-human Alexa 488 antibody. **B)** Heat map showing results from one representative experiment. In the presence of control mIgG2a no loss of mB7-1 hIgG1 binding was observed. The graph shows the average and standard deviation for data from three independent experiments. This data was fit using a one-site competition model equation in the software Prism and the calculated EC_50_ was 8.3 ± 1.5 nM.
(PDF)

S9 Fig Expression, purification and testing of recombinant PD-L1 proteins used for T-cell activation assays. Top panel: A coomassie stained SDS/PAGE gel showing the recombinant Fc-fusion proteins that were purified over nickel affinity resin and gel filtration. Lower panel: FACS scatter plots for a protein binding experiment. Cells transiently expressing either mPD-1 or mB7-1 as GFP fusions were challenged with purified recombinant protein as shown. Protein binding was detected using an anti-mouse Alexa 594 secondary antibody followed by FACS analysis. These data demonstrate that the purified recombinant proteins maintain the same binding phenotype.
(PDF)

S10 Fig Gel Filtration of recombinant PD-L1 proteins used for T-cell activation assays. **A) Left** Analytical gel filtration traces for small-scale purified recombinant WT PD-L1-mIgG2a, PD-1-mIgG2a and B7-1-mIgG2a proteins. **Right** Analytical gel filtration traces for select PD-L1 mutants used for the T-cell activation experiments. **B)** S200 size exclusion chromatography of nickel affinity purified WT PD-L1-mIgG2a from a large-scale 600mL HEK 293 suspension culture. **C)** Same as **B)** but for a subset of PD-L1 mutants with selective binding for either PD-1 (G119D and G120D) or B7-1 (D122A and R125A).
(PDF)

S11 Fig Reexamination of mPD-L1 Fc-fusion proteins by analytical gel filtration. A) Analytical Size exclusion for fresh preparations of WT PD-L1, Y56A, G119D, G120D, D122A and R125A (inset of gel shows eluates off His60 resin) from 150mL of HEK suspension cells. VPA was added at 24hours, culture supernatants were collected on day 7 and purified over His60 resin. Nickel eluates were concentrated to 500uL and loaded onto the Superose 6 Increase column 10/300. The void for this column runs at 8.5mL. The peak containing dimer Fc-fusion is found ~14.8mL and was collected. There was also a small peak at ~11mL and a shoulder peak at ~ 13mL. B) Analytical Size exclusion was re-run 2 weeks later for WT PD-L1, Y56A, G119D, G120D, D122A and R125A. Eluates collected from the initial size exclusion run were stored at 4C for 2 weeks, concentrated 2X and 100uL was loaded onto the same analytical column. The predominant peak for all of the proteins remains around 14.8mL. There is no longer a peak at 11mL though a more defined peak is observed at ~13mL (previously where the shoulder peak was). This data suggests the PD-L1 Fc fusion protein is reasonably stable and the mutants show some deviation but behave very similarly to wild-type protein.
(PDF)

S12 Fig Staining isolated CD^+^ T-cells for PD-1 and B7-1 expression. CD^+^ T-cells were isolated as described in the methods and plated in wells of a 96-well plates in the absence (TOP) or presence (BOTTOM) of 5ug anti-CD3. After 4-days cells were washed and incubated either with anti-human 647 secondary antibody alone (BLUE) or with primary anti-mPD-1 or anti-mB7-1 antibodies (human IgG1 from R&D Systems) as indicated. After staining cells were analyzed by flow cytometry and live 647 (APC) positive cells were gated based on the secondary antibody alone controls for each condition.
(PDF)

S13 Fig mB7-1 mutants express to a similar extent as wild-type mB7-1. Graph shows the %mCherry positive HEK 293 cells transfected with wild-type mB7-1, mutant B7-1 or mCherry empty vector control. Data is the average from three independent transfections with error bars showing the standard deviation.
(PDF)

S14 Fig mPD-L1 and mCTLA-4 binding to mB7-1 IgC mutants. Graph highlighting the panel of 40 B7-1 IgC mutants examined for binding to mPD-L1 (Red Bars) and mCTLA-4 (Gray Bars) as described in the main text. Data shows percent bound from three independent experiments with error bars representing the standard deviation.
(PDF)

S15 Fig Saturation binding of mPD-L1 and mCD28 to beads coated with mB7-1. Data shows saturation binding curves used to determine the lowest concentration of either mPD-L1 hIgG1 or mCD28 hIgG1 protein to saturate protein A beads loaded with mB7-1 mIgG2a. For competition experiments, 5nM was added to B7-1 loaded beads in the presence of increasing concentrations of the competing proteins (mIgG2a control, CTLA-4 mIgG2a, CD28 mIgG2a, mPD-L1 mIgG2a).
(PDF)

S16 Fig mB7-1 expressing cells do not bind mPD-L1 expressing cells. **A)** Representative scatter plots from cell-cell conjugation experiment. HEK 293 suspension cells expressing full-length mPD-1 and mB7-1 mCherry fusions were mixed 1:1 with cells expressing either mCTLA-4 GFP, mPD-L1 GFP or GFP control. Positive binding between the populations of cells is observed as an increase in the percentage of events in Q2 (upper right). **B)** Quantification of three independent cell-cell conjugation experiments. Data shows the average and standard deviation.
(PDF)

S17 Fig Cells co-overexpressing B7-1 and PD-L1 DO NOT cause loss of CIS mB7-1 SmBit and mPD-L1 LgBit dependent luminescence. A) This experiment was setup similarly to that shown in Fig 7B. Cells expressing different SmBit/LgBit combinations as indicated were titrated with cells co-expressing B7-1 mCherry, PD-L1 GFP (solid lines) or with cells expressing CTLA-4 mCherry (dashed lines). No change in luminescence is observed with cells overexpressing B7-1 or PD-L1 suggesting the luminescence signal is not being driven by trans B7-1/PD-L1 binding. In contrast addition of CTLA-4 expressing cells results in a significant increase in B7-1 luminescence and a significant decrease in B7-1/PD-L1 cis luminescence. This is similar to what we demonstrate in the manuscript. The data represents three independent experiments.
(PDF)

S1 Table Cell microarray analysis of PD-L1 mutant binding to PD-1 and B7-1. The table shows scores for binding from the analysis of cell microarrays printed with PD-L1 mutants as shown in Fig 1E where “**+**” signifies a fluorescent intensity in the 647 channel (bound protein) comparable to WT, “**−**”signifies no detectable fluorescence and “**R**” signifies reduced fluorescent intensity compared to WT PD-L1. Scores reflect observations made from three independent experiments. Mutants labeled **BLUE** lost binding to both PD-1 and B7-1, those in **RED** lost binding only to B7-1 and those in **GREEN** lost binding only to PD-1.
(PDF)

S2 Table Flow cytometry microbead binding analysis of PD-L1 mutant binding to PD-1 and B7-1. The table shows the analysis of PD-1 and B7-1 binding to a subset of PD-L1 mutants as determined by flow cytometry as shown in Fig 2. Data is the calculated average and standard deviation from three independent experiments normalized to WT PD-L1 binding. Mutants labeled in **RED** lost binding only to B7-1 and those in **GREEN** lost binding only to PD-1. Mutants left black showed a reduction in both PD-1 and B7-1 binding.
(PDF)

S3 Table Data from the analysis of PD-L1, CTLA-4 and CD28 binding to B7-1 mutants determined by flow cytometry. The table shows the analysis of B7-1 mutant binding to PD-L1, CTLA-4 and CD28 as determined by flow cytometry in Fig 4. Data shows the average and standard deviation from three experiments. Boxes highlighted **RED** show >20% binding comparable to WT B7-1 and boxes highlighted **YELLOW** show 20–50% binding comparable to WT B7-1.
(PDF)
