# Supplementary material for: Mechanistic dissection of the PD-L1:B7-1 co-inhibitory immune complex
Source: PLoS One. 2020 Jun 4;15(6):e0233578. doi: 10.1371/journal.pone.0233578 (PMC7272049; doi:10.1371/journal.pone.0233578)
Supplement: S1 Method — (PDF) [file pone.0233578.s001.pdf]

SUPPLEMENTAL INFORMATION

Supplemental Methods

Vector Design, IgSF Library Cloning & Expression Validation

The Ig superfamily (IgSF) library was constructed using a ligation independent cloning (LIC) vector that was designed such that full-length ectodomains of each IgSF member could be inserted between an EPO leader peptide and the PD-L1 transmembrane domain. This construct utilizes a codon optimized human Erythropoietin leader sequence for efficient secretion and ER processing, immediately followed by a 5' Ligation independent cloning (LIC) site and a *Bacillus subtilis* levansucrase (SacB) protein for negative selection during high-throughput cloning. Positioned 3' to the LIC site is the native PD-L1 transmembrane helix, followed by a covalently linked mCherry. Templates for cloning were obtained from the human mammalian genome collection (MGC) cDNA set from OpenBiosystems or synthesized (Genscript; genscript.com). The full-length ecto domain sequence for each IgSF target was determined using the structural boundaries predicted by Signal P and TMHMM, which use sequence-based prediction algorithms to identify leader peptides and transmembrane domains, respectively (48, 49). PCR primers added the appropriate overhangs to the 5' ends (Forward: 5' CCTCTCGGCCTGCCTGTGCTAGGCNNN 3' Reverse: 5' CCGCTGCCGCGTGGGACGAGNNN 3'). Standard PCR amplification was performed using KOD polymerase (EMD Biosciences). We did not anticipate that tethering secreted proteins to the membrane surface would significantly impact their binding functions. To validate the library prior to screening, the individual library components were transfected into 1mL suspension adapted HEK293 cells in arrayed 24-well tissue culture plates. After 72 hours post transfection cell surface localization was evaluated by fluorescent microscopy and scored manually (0-3) based on mCherry fluorescence intensity and correct plasma membrane localization of mCherry (Fig. S2). This stategy resulted in the generation of 196 sequence and expression validated constructs of members of human IgSF and TNFRSF. Of 280 constructs cloned only those showing strong expression (scored as 2 or 3) and correct membrane localization were utilized for further analysis (196 constructs in total, ~71%). For convenience in testing the cell microarray platform, we limited the printed library to 144 of the 200 expression-validated clones.

PD-L1 & B7-1 Site-directed Mutagenesis Cont.

For PD-L1, positions selected for mutagenesis were based on the crystal structure of complex formed by human PD-L1 and murine PD-1 (PDB: 3BIK). Equivalent surface accessible positions

in mouse PD-L1 were identified by primary sequence alignment to human PD-L1 (36 positions total from the IgV domain, and 42 in IgC domain). Mutagenesis was attempted such that each chosen position was mutated to an Ala, Glu or Arg residue. The overall mutagenesis success rate was ~78%, and for some positions not all substitutions (A, E and R) were obtained. The sequence validated mutants were expression tested by transient transfection of 1 mL of suspension HEK 293 cells. Only those mutants exhibiting comparable expression to wild type PD-L1 and correct plasma membrane localization were subsequently utilized in the microarray and FACS binding studies, yielding a final library of 125 PD-L1 mutants to assay.

Residues of mB7-1 were selected for mutagenesis by first determining the surface accessible residues in human B7-1 (PDB: 1DR9) followed by a primary sequence alignment with mB7-1 to determine the equivalent residues. A total of 60 residues in the IgV domain and 46 residues in the IgC domain were mutated to an Ala or Glu except for all acidic residues which were mutated to Arg with an overall cloning success rate of 73% (155 mutants total). Of the sequence-validated set 149 mutants showed expression and localization similar to the wild-type mB7-1 mCherry protein and were selected for binding analysis. For mapping experiments, 0.5 µg of soluble mCTLA-4 Fc, mCD28 Fc or mPD-L1 Fc (generated in house as described below) was added to 100,000 HEK 293 suspension cells transiently transfected with either wild-type of mutant B7-1 arrayed in 96-well V-bottom plates. After binding for 1 hour at room temperature, cells were washed by pelleting at 500xg for 5min and resuspending in 1X PBS with 0.2% BSA. Following one additional wash, 0.25 µg of anti-mouse Alexa 488 (Invitrogen) was added and cells incubated for 30 min at room temperature. After antibody binding, cells were washed three more times and analyzed by FACS for binding as described above.

Purification of Recombinant Fc-fusion protein

To clone mPD-L1 Fc-fusion proteins, full-length wild type or mutant PD-L1 ectodomains (residues F19 - R237) were sub-cloned into a LIC vector containing a C-terminal decahis-tagged Fc domain (mIgG2a-His_{10}). In addition, wild type mPD-1 Fc (mPD-1 residues L25 - Q167), mB7-1 Fc (mB7-1 residues D37 - K245), mCTLA-4 Fc (mCTLA-4 residues A37 – F162), and mCD28 Fc (mCD28 residues K21 – K149) constructs were also cloned both as mIgG2a-His_{10} and hIgG1-His_{10} fusions. All expression constructs as well as mIgG2a and hIgG1 isotype control constructs were transiently expressed in 1.2L of HEK 293 suspension cells. Six days post transfection, the media was harvested, 50 mM MES was added to adjust to pH 6.5 and 100 mM Arg-Cl (pH 6.5) was added to enhance solubility and hinder aggregation. Fc-fusions were

subsequently purified by Ni^{2+}-NTA chromatography (GE) using a batch binding method (10 mL resin bed volume) followed by gravity flow over a glass column. The Ni^{2+}-NTA resin was washed with 50 column volumes of wash buffer (50 mM MES pH 6.5, 100 mM Arg-Cl, 5 mM imidazole, 150 mM NaCl, 10% Glycerol) and the bound protein eluted with 50 mL the same buffer containing 500 mM imidazole. Eluates were concentrated and further purified by gel filtration on an S200 sephadex column (GE) equilibrated with 50 mM MES pH 6.5, 100 mM Arg-Cl, 150 mM NaCl, 10% Glycerol. All recombinant proteins for use in T-cell activations experiments were used within one week of purification. For biochemical assays and in vitro HEK cell binding experiments frozen aliquots of protein were utilized and routinely checked for potential aggregation by analytical size chromatography.

Cell Microarray Printing and Transfection

For array generation, concentrated miniprep plasmid DNA (eluted in ultra-pure H_2O and normalized to 200ng/μL) was diluted to 100ng/μL (1:1) with a 0.38% gelatin solution (final gelatin 0.19%) in 96-well PCR plates. The gelatin solution was prepared by dissolving 0.38g of gelatin in 100 mL ultra-pure H_2O at 60°C for 1-2 hours and filtering through a 0.44um filter prior to storage. Before use, the gelatin solution was warmed to 60°C for 1 hour. After mixing in 96-well, plates, the plasmid DNA/gelatin mixtures were rearrayed into 384-well plates for printing. The remaining DNA/gelatin mixture could be stored at 4°C for up to 2 weeks. For printing, poly-L-lysine coated glass microscope slides (polysciences #22247) were pre-washed in sterile water for 1 hour at room temperature and spun dry in 50mL conical tubes. The Microarray printer at the Einstein cDNA Microarray Facility is a custom designed robot capable of printing 230 arrays at a time with density of over 36,000 spots per slide with a precision of 5 microns (50). The robot is surrounded by a soft-wall enclosure providing HEPA-filtered air with temperature and humidity control. The cell microarrays were printed using SMT-S150 silicon pins from Parallel Synthesis Technologies, Inc., which produce spots with a diameter of 150-200 microns. The dot pattern selected was a 12 X 12 grid using 4 pins for a total of 576 spots. The environment was maintained at a temperature of 22^0C and 75% relative humidity for uniform spot size. Mild sonication was used to clean the pins between pick-ups to minimize both cross-contamination and pin clogging from the viscous media.

Printed slides were stored at 4°C in a desiccated chamber. For transfection of the printed slides, HEK EBNA cells were grown in 15 cm plates to about 80% confluence. Media was removed and cells were washed twice with 1x PBS. Fresh media was used to displace and collect EBNA cells

from the plate by gentle pipetting. The cells were counted and adjusted to $2x10^6$ cells/mL. Hybriwell chambers (Grace Bio-labs #612101) were placed over printed slides and 3 slides total placed into a square petri dish (non-TC plate). Effectene transfection reagent (Qiagen) was prepared for each slide by diluting 16 µL of the Enhancer solution into 150 µL of the EC Buffer, mixing briefly and subsequently adding 25 µL of the Effectene reagent. This solution was immediately pipetted into the Hybriwell port covering the entire surface of the slide and incubated for 20 min at room temperature. After incubating, Hybriwells were removed and 10 mL of the prepared HEK EBNA cell suspension was added to the dish such that all three slides were evenly covered. Cells were allowed to adhere for 1 hour at 37°C in a CO_2 incubator, after which the media and any unattached cells were removed and replaced with 10 mL of fresh media. Slides were then incubated for 3 days at 37°C in a CO_2 incubator to allow for monolayer formation and transfection to occur.

Cell Microarray Binding and Analysis Cont.

Cell microarrays were challenged with 150 µL of 100 nM Fc-fusion protein pre-incubated with anti-human (H+L) Alexa 647 secondary antibody (Invitrogen 1/100 dilution). All Fc-fusions used to query the protein cell microarrays were purchased from R&D systems. This solution was pipetted drop wise over the surface of the transfected slide and a lifterslip coverslip (Thermo #25X60I24789001LS) was placed on top to help evenly distribute the protein mixture across the slide. Slides were covered with foil and incubated at room temperature for 30 min. After binding, 1x PBS and 0.2% BSA was used to float the lifterslips off each slide, after which the slides were washed once with 1X PBS and 0.2% BSA, followed by two washes with 1X PBS. After washing, slides were fixed in 1X PBS containing 4% formaldehyde for 10 minutes at room temperature and washed twice with 1X PBS. A coverglass was placed over the treated slides for protection. Slides were scanned on a Genepix 4400A microarray scanner using the Cy3 (mCherry fluorescence) and Cy5 (Alexa 647 fluorescence) filters. The PMT and laser power settings were adjusted for each slide such that signals were optimized so that they were not saturating and also so that the signals from each channel (Cy3 and Cy5) were within the same intensity range with respect to each other. Depending on the experiment, the PMT was typically set between 550 - 700 and the laser power set to between 80-90%.

Microbead FACS Binding Assay Cont.

For each experiment, 500uL of protein A microbeads were loaded with a mixture of 10 µg FITC-Fc and 40 µg of either our purified mPD-1-Fc or mB7-1-Fc protein in a total volume of 5mL 1x

PBS. The beads were incubated overnight (~16 hours) at 4°C. Loaded beads were stored for up to two weeks prior to use. Initial experiments determined that 75 µL of the loaded beads were sufficient to saturate 150,000 cells transfected with wild type PD-L1, (transfection efficiency being consistently 60-70%). For titration experiments, sets of wild type and mutant PD-L1 constructs were transfected in 24-well tissue culture plates containing 1mL of suspension HEK-293 cells. Three days post transfection cells were counted and diluted to $1x10^6$ cell/mL with 1x PBS with 2% BSA. 150K cells (150uL) were transferred to Eppendorf tubes, and 75uL of loaded microbeads added along with an additional 100uL 1x PBS with 2% BSA. Reactions were mixed end over end for 1 hour at 4°C, DAPI was added and samples were immediately analyzed by flow cytometry on a BD Aria III cytometer. Data were analyzed by gating first for live cells (DAPI negative) then for mCherry positive cells (PD-L1 expression). The percentage of mCherry positive cells that were FITC positive (microbeads bound) was used as “percent bound”. For each experiment, the percent bound was normalized to wild type binding.

FACS Titration Assay

FACS titration assays were performed with PD-1 Fc and B7-1 Fc fusion proteins purified as described above. HEK 293 suspension cells were transfected with the wild type or mutant PD-L1 constructs. Three days post transfection cells were counted and diluted to $1x10^6$ cells/mL in 1x PBS. Premixed solutions containing a final concentration of 1 uM Fc-fusion protein and 1.5 uM Alexa 488 goat anti-mouse secondary antibody were incubated on ice for 30 min. Subsequently, increasing amounts of the premixed solution was added to wells of a 96-well plate and the volume adjusted to 50uL with 1x PBS and 150 µL of diluted cells (150,000 cells total) added to the wells. Binding was performed at 4°C for 1 hour, the cells washed 3x with 1x PBS by centrifugation and subsequently analyzed by FACS. Gated live cells were sub-gated for mCherry, and mCherry positive cells sub-gated for Alexa-488. The percent bound represents the percentage of mCherry cells that were Alexa-488 positive. Data points represent the average of three independent experiments fit to the single site binding equation: $Y = B_{max}*X/(EC_{50} + X)$.

PD-1/B7-1 Bead Competition Binding Experiment

For bead loading, mPD-L1 mIgG2a (50 µg) was added to 5 µL protein A beads (Bangs Labs) in 25 µL total volume and incubated for 30 minutes at room temperature. After binding the beads were washed twice with 250 µL of 1X PBS and 0.2% BSA by centrifugation at 500xg. For binding experiments, 5 µL of diluted beads (~ 0.1 µL total) were added to increasing

concentrations of mB7-1 hIgG1 protein (0 – 50 nM) in a final volume of 100 µL 1X PBS and 0.2% BSA. For competition experiments, 5 µL of PD-L1 loaded beads and 5 nM mB7-1 hIgG1 were added to increasing concentrations of PD-1 mIgG2a protein (0.01 – 250 nM). Parallel experiments were carried out in which purified mIgG2a isotype control was titrated at equivalent molar concentrations. All protein binding was performed in 96-well plates at 22°C, with shaking at 900 rpm for 1 hour. After binding, plates were washed two times with 1X PBS with 0.2% BSA, and anti-human (H + L) Alexa 488 labeled secondary antibody (Invitrogen) was added at 0.01 µg/µL (1 µg total) and incubated for 30 min. Beads were subsequently washed two more times with 1X PBS with 0.2% BSA. Samples were analyzed by flow cytometry and the data gated for beads that were Alexa 488 positive (FL1 – B7-1 Binding). Competition data was normalized to 5 nM B7-1 binding in the absence of mPD-1 and plotted as a function of log [mPD-1]. Average data from three independent experiments were fit to a one-site competition model equation: $Y = min + (max – min) / (1 + 10^{x-logEC50})$.

PD-L1/CTLA-4/CD28 Bead Competition Binding Experiment

Protein A beads were loaded as described above with mB7-1 mIgG2a protein. For the first set of competition experiments mPD-L1 hIgG1 (5 nM, R & D Systems) was added to 5 µL mB7-1 loaded beads and incubated with increasing concentrations of either mCTLA-4 mIgG2a, mCD28 mIgG2a or mIgG2a isotype control (0 – 250 nM). In another set of competition experiments mCD28 hIgG1 (5 nM, R & D Systems) was added to mB7-1 loaded beads and titrated with mCTLA-4 mIgG2a, mPD-L1 mIgG2a or mIgG2a isotype control (0 – 250 nM). All experiments were carried out and analyzed as described above for PD-1/B7-1 competition.

cis and TRANS Competition Experiments Using Split NanoLuciferase

For co-transfection competition experiments, 50ng of empty mCherry N1 vector, WT mPD-L1 mCherry, WT mB7-1 mCherry or specific mB7-1 mCherry point mutant DNA was co-transfected with mB7-1 SmBit (225ng) and mPD-L1 LgBit (225ng). The resulting transfected cells were then analyzed as described above. For trans competition experiments with mPD-1, mCTLA-4 and mCD28 recombinant protein, HEK 293S cells were transfected with the stated SmBit and LgBit construct combinations. Two days post transfections cells were counted and 1 µg of recombinant mPD-1 hIgG1, mCTLA-4 hIgG1 or hCD28 hIgG1 was added to 100,000 cells in a final volume of 150uL in 96-well V-bottom plates. Protein was allowed to bind for 1 hour with shaking at 900rpm at room temperature. As described above, a 50 µL aliquot from each reaction was removed for luminescence detection. Similarly, for trans competition experiments

with mPD-1, mCTLA-4 and mCD28 expressing cells, on the same day as the transfection of split nanoluciferase pairs, separate transfections were setup for GFP N1 empty vector, mPD-1 GFP, mCTLA-4 GFP and mCD28 GFP. For binding, 50,000 GFP transfected cells were added to 100,000 SmBit/LgBit transfected pairs as indicated and allowed to bind for 1 hour with shaking at 900rpm at room temperature. A 50 μL aliquot from each cell binding reaction was removed for luminescence detection.
