# Supplementary figures and images for: Mechanistic dissection of the PD-L1:B7-1 co-inhibitory immune complex
Source: PLoS One. 2020 Jun 4;15(6):e0233578. doi: 10.1371/journal.pone.0233578 (PMC7272049; doi:10.1371/journal.pone.0233578)

Figure S1

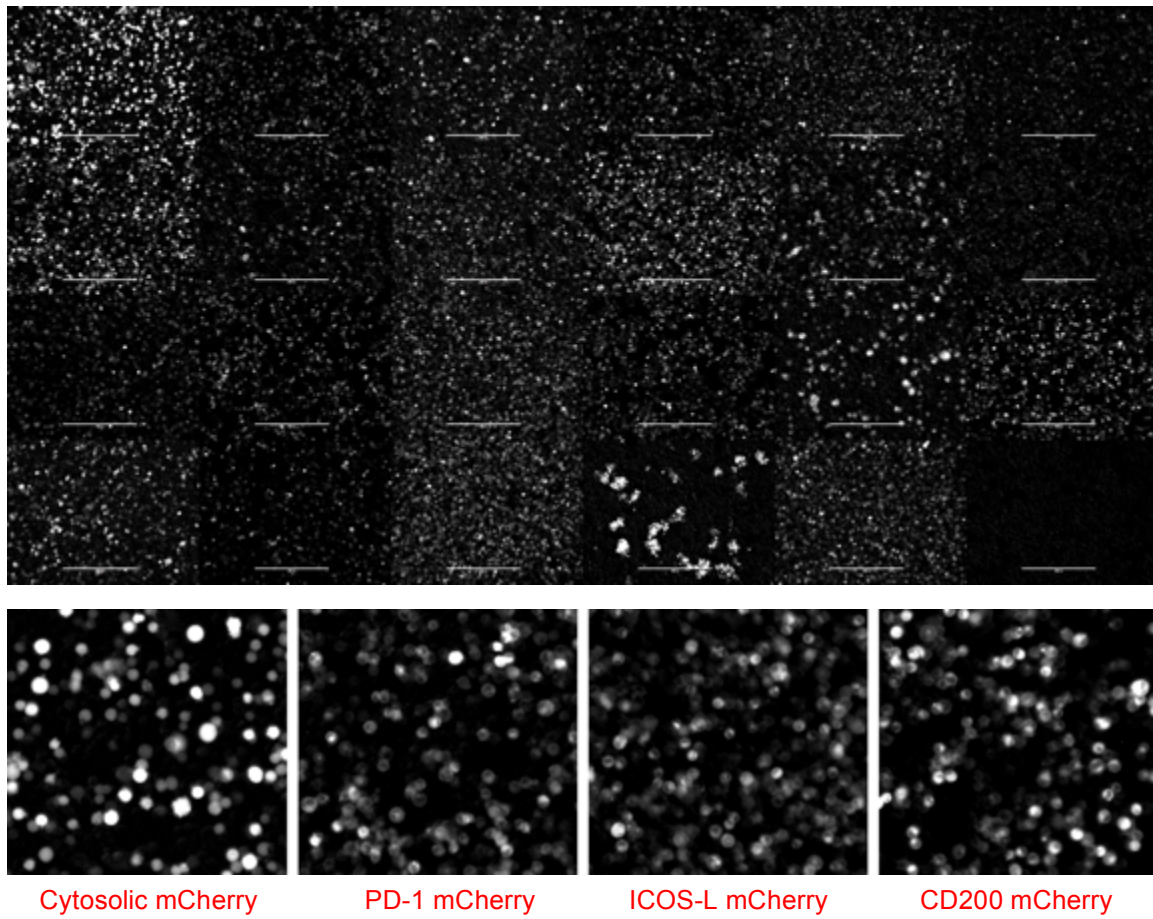

Supplement: S1 Fig — The top panel shows 24 representative images of HEK 293 cells expressing different Ig superfamily targets in our Type I mCherry vector. All images were acquired on a EVOS inverted benchtop florescence microscope. Cytosolic mCherry was transfected as a negative control for membrane localization (Top Left rectangle) and the bottom right most rectangle shows untransfected cells. The bottom panel shows a 10X zoomed images of select constructs depicting the difference observed between cytosolic and membrane localized constructs. (PDF) [file pone.0233578.s002.pdf]

Figure S2

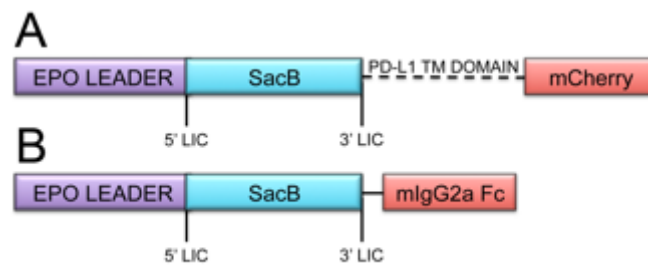

Supplement: S2 Fig — A) We employed ligation independent cloning (LIC) methods for the design of custom vectors in which the SacB killer gene is replaced by the full-length ectodomain of a gene of interest flanked by on the N-terminus by the leader sequence from the human erythropoietin gene and flanked on the C-terminus by the transmembrane domain from mouse PD-L1 followed by mCherry fluorescent protein. B) The same LIC sites (and therefore the same PCR products) can be used to clone into a separate vector for the expression of Fc fusion proteins for downstream validation experiments. (PDF) [file pone.0233578.s003.pdf]

Figure S3

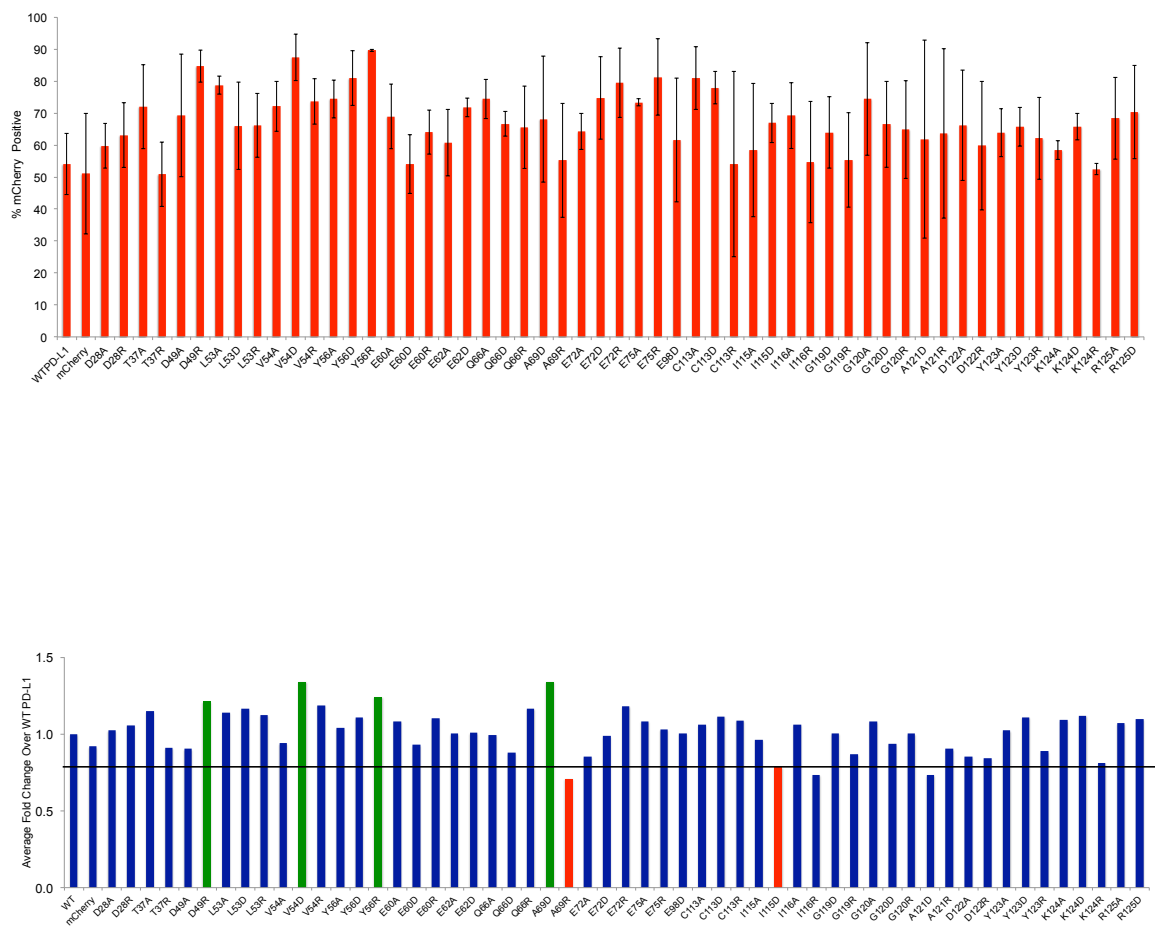

Supplement: S3 Fig — TOP Graph shows the %mCherry positive HEK 293 cells transfected with wild-type PD-L1, mutant PD-L1 or mCherry empty vector control. Data is the average from three independent transfections with error bars showing the standard deviation. BOTTOM One-way ANOVA analysis was performed to determine statistically significant differences between each mutant compared to WT PD-L1. To aid in visualizing the results of this analysis, the graph shows the fold change in average expression for each mutant compared to wild-type PD-L1 (normalized to 1). All of the mutants shown in BLUE were not statistically different from wild-type, those in GREEN were significantly different but showed higher expression than WT, those in RED were significantly different and showed ~25% less expression than WT. (PDF) [file pone.0233578.s004.pdf]

Figure S4

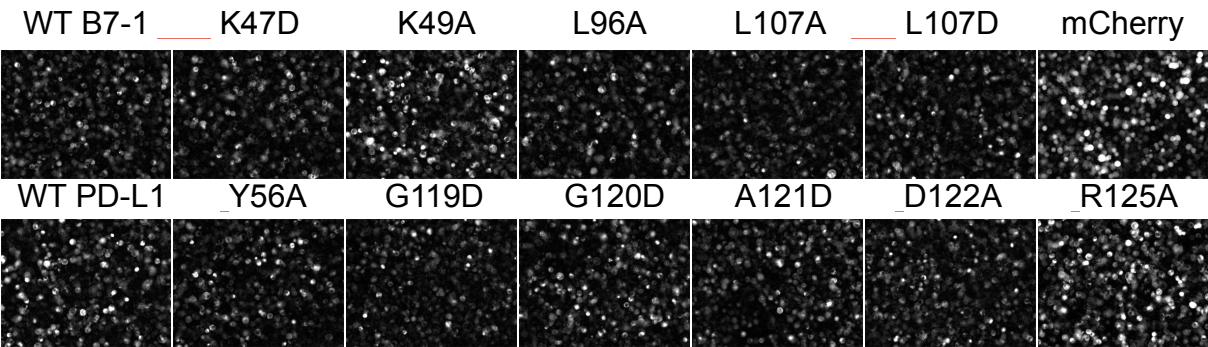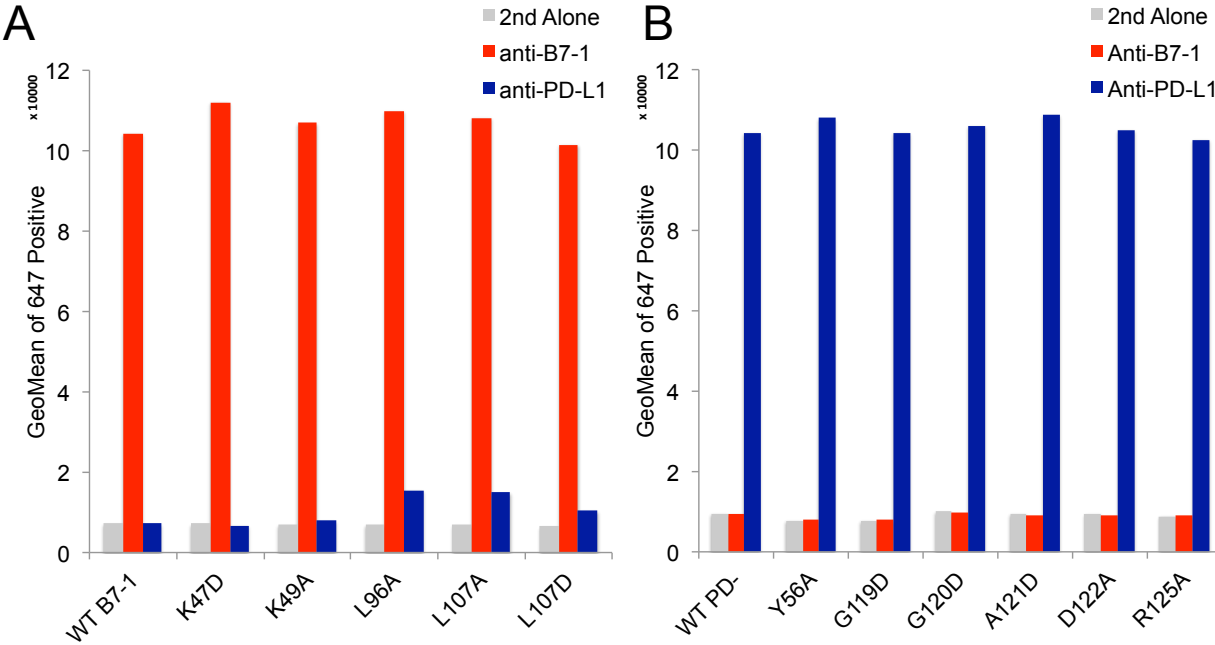

Supplement: S4 Fig — TOP HEK 293 suspension cells were transiently transfected with either wild type or mutant mPD-L1 or mB7-1 as indicated in 24-well suspension plates. Two days post transfection cells were imaged for mCherry expression using an EVOS inverted benchtop florescence microscope. BOTTOM HEK 293 suspension cells were transiently transfected with either wild type or mutant mPD-L1 or mB7-1 as indicated. Two days post-transfections, 100,000 cells from each transfection were incubated with 0.5ug of each monoclonal antibody (R&D Systems MAB90783 (anti-mPD-L1) and R&D Systems MAB740 (anti-mB7-1) for 1 hour with shaking at room temperature. Cells were subsequently washed three times with 1X PBS with 0.2% BSA and incubated with secondary antibodies (anti-Rabbit 647 (PD-L1) and anti-Rat 647 (B7-1). Cells were analyzed by flow cytometry and data presented as the GeoMean of 647 (bound). (PDF) [file pone.0233578.s005.pdf]

Figure S5

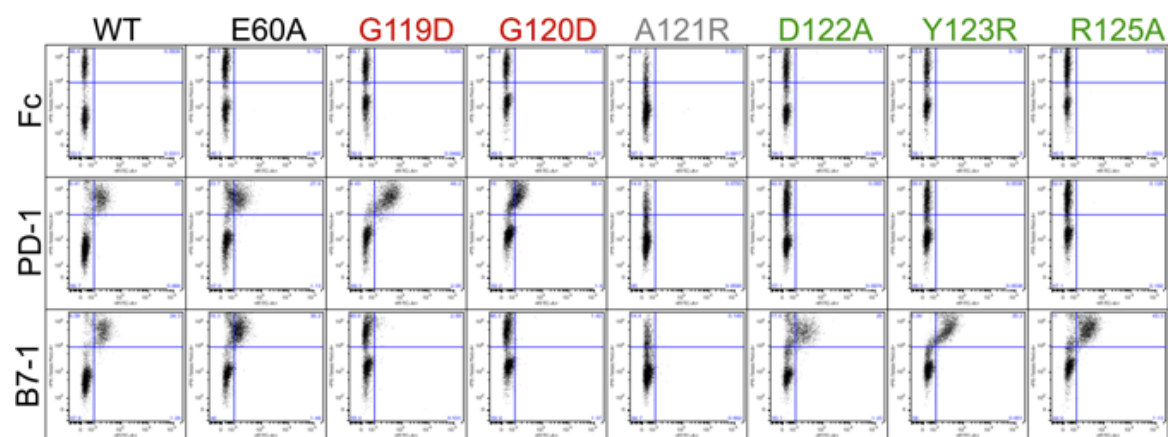

Supplement: S5 Fig — Data shows a representative set of FACS scatter plots obtained from the microbead binding experiment. Microbeads coated with either control, PD-1 or B7-1 Fc-fusion protein were used to challenge cells expressing wild-type PD-L1 or mutants. The E60A mutant did not affect binding of PD-L1 to either PD-1 or B7-1. G119D and G120D lost binding to B7-1 but maintained binding to PD-1. The A121R mutant does not bind either PD-1 or B7-1. The D122A, Y123R and R125A mutants all maintained binding to B7-1 but lost binding to PD-1. (PDF) [file pone.0233578.s006.pdf]

Figure S6

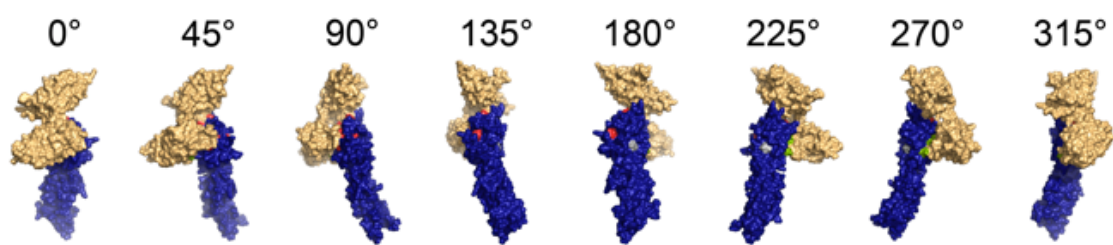

Supplement: S6 Fig — 360 degree rotation of a space filling representation of the PD-1:PD-L1 crystal structure (PDB: 3SBW). Residues are color coded the same as previously described (Green = PD-1 binding null, Red = B7-1 binding null, Gray = Both null). Most of the PD-1 specific residues are buried at the interface within the complex and therefore not visible. In contrast, many of the B7-1 residues remain exposed in the space fill model demonstrating that these positions are not involved in and do not impact the PD-1 binding interface. (PDF) [file pone.0233578.s007.pdf]

Figure S7

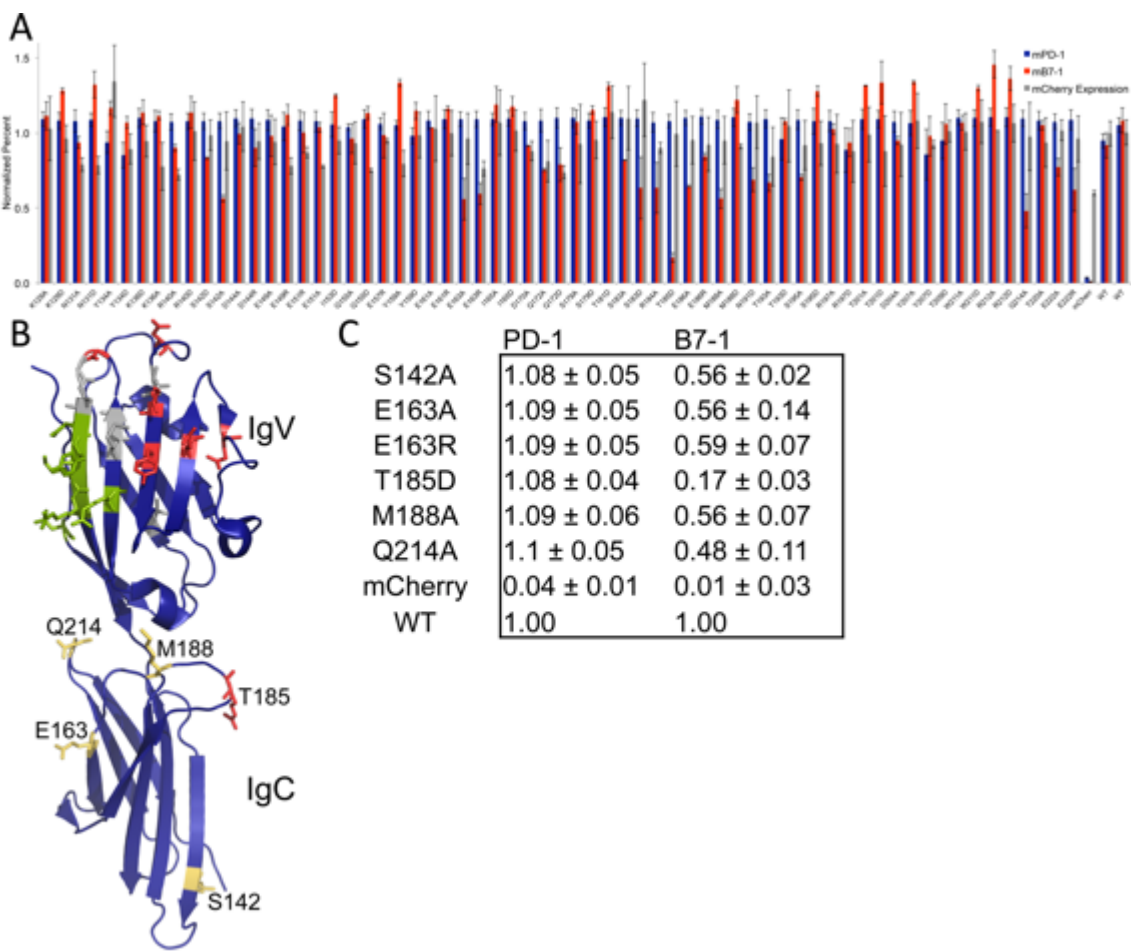

Supplement: S7 Fig — A) A panel of 65 PD-L1 IgC mutants were examined for binding to mPD-1 (Blue Bars) and mB7-1 (Red Bars) using the microbead binding assay described in the main text. Gray bars depict the %mCherry expression for each mutant normalized to wild-type. All data represents two independent experiments with error bars showing the standard deviation. B) Mapping of the IgC mutants onto the structure of PD-L1 (PDB: 3SBW). In the IgV domain the color coding is the same as the main text, green = PD-1 binding affected, red = B7-1 binding affected, gray = both PD-1 and B7-1 binding affected. For the IgC domain T185D showed the most significant effect on B7-1 binding, highlighted red while the other mutants identified showed more modest effects, highlighted yellow. C) Table showing the normalized average binding of PD-1 and B7-1 to these select mutants. (PDF) [file pone.0233578.s008.pdf]

Figure S8

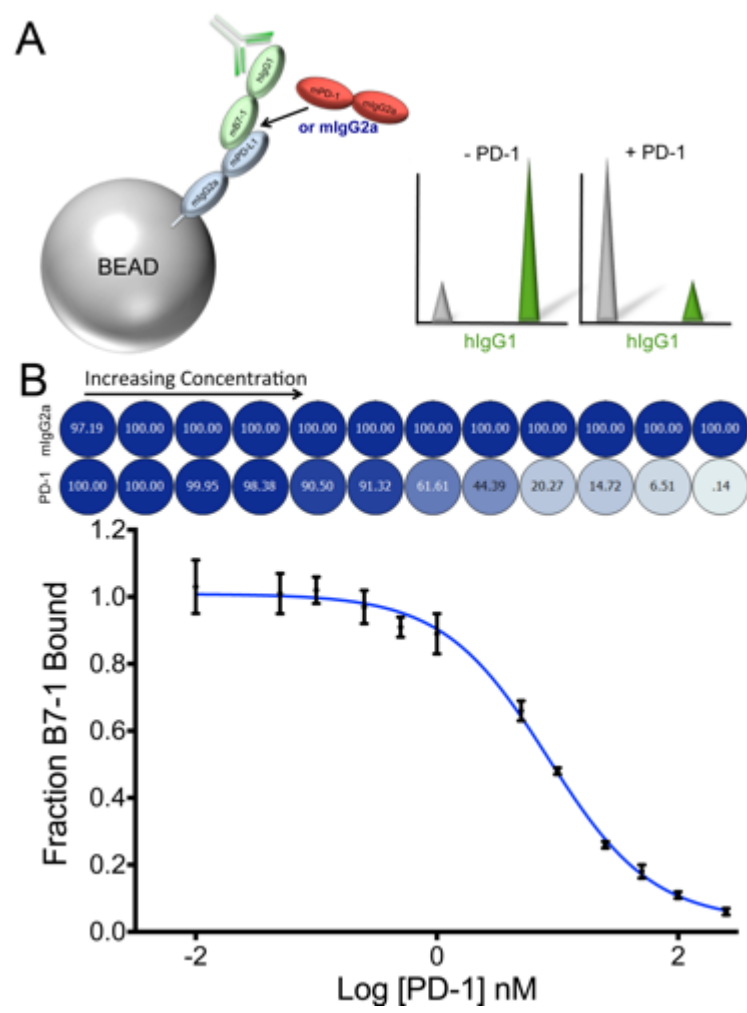

Supplement: S8 Fig — A) Cartoon depiction of the competition assay. Briefly, protein A beads were saturated with mPD-L1 mIgG2a protein and subsequently incubated with 20nM mB7-1 hIgG1 and an increasing concentration of either mIgG2a, mPD-1 mIgG2a. Binding of mB7-1 hIgG1 was determined using an anti-human Alexa 488 antibody. B) Heat map showing results from one representative experiment. In the presence of control mIgG2a no loss of mB7-1 hIgG1 binding was observed. The graph shows the average and standard deviation for data from three independent experiments. This data was fit using a one-site competition model equation in the software Prism and the calculated EC50 was 8.3 ± 1.5 nM. (PDF) [file pone.0233578.s009.pdf]

Figure S9

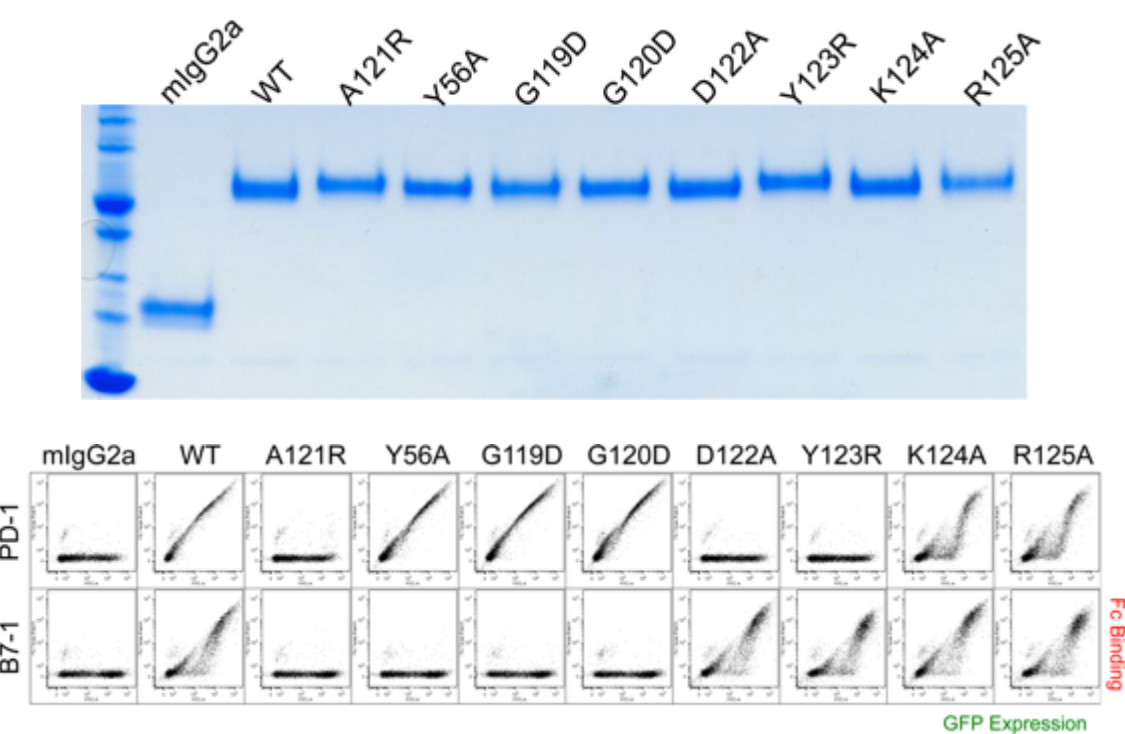

Supplement: S9 Fig — Top panel: A coomassie stained SDS/PAGE gel showing the recombinant Fc-fusion proteins that were purified over nickel affinity resin and gel filtration. Lower panel: FACS scatter plots for a protein binding experiment. Cells transiently expressing either mPD-1 or mB7-1 as GFP fusions were challenged with purified recombinant protein as shown. Protein binding was detected using an anti-mouse Alexa 594 secondary antibody followed by FACS analysis. These data demonstrate that the purified recombinant proteins maintain the same binding phenotype. (PDF) [file pone.0233578.s010.pdf]

Figure S10

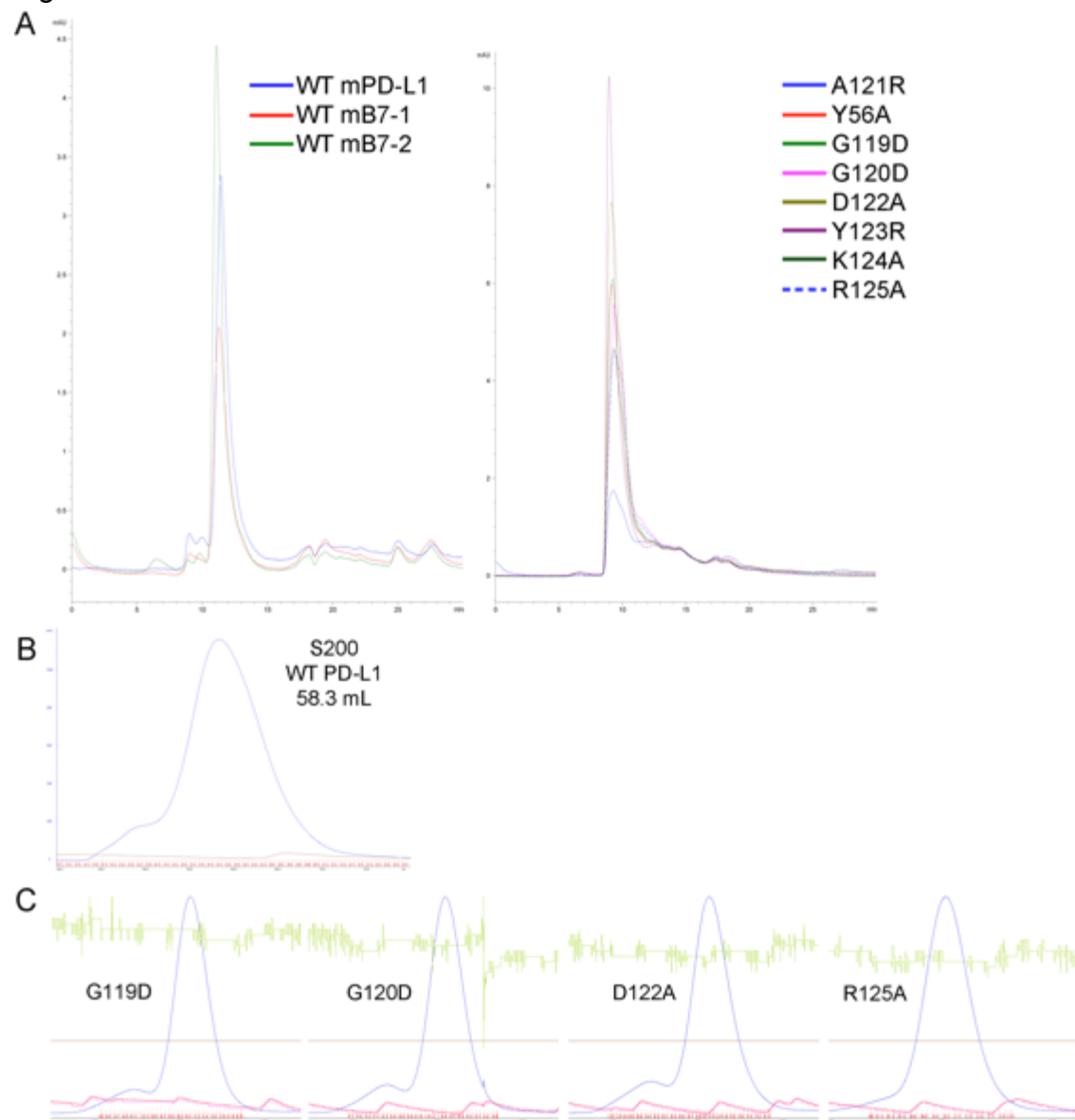

Supplement: S10 Fig — A) Left Analytical gel filtration traces for small-scale purified recombinant WT PD-L1-mIgG2a, PD-1-mIgG2a and B7-1-mIgG2a proteins. Right Analytical gel filtration traces for select PD-L1 mutants used for the T-cell activation experiments. B) S200 size exclusion chromatography of nickel affinity purified WT PD-L1-mIgG2a from a large-scale 600mL HEK 293 suspension culture. C) Same as B) but for a subset of PD-L1 mutants with selective binding for either PD-1 (G119D and G120D) or B7-1 (D122A and R125A). (PDF) [file pone.0233578.s011.pdf]

Figure S11

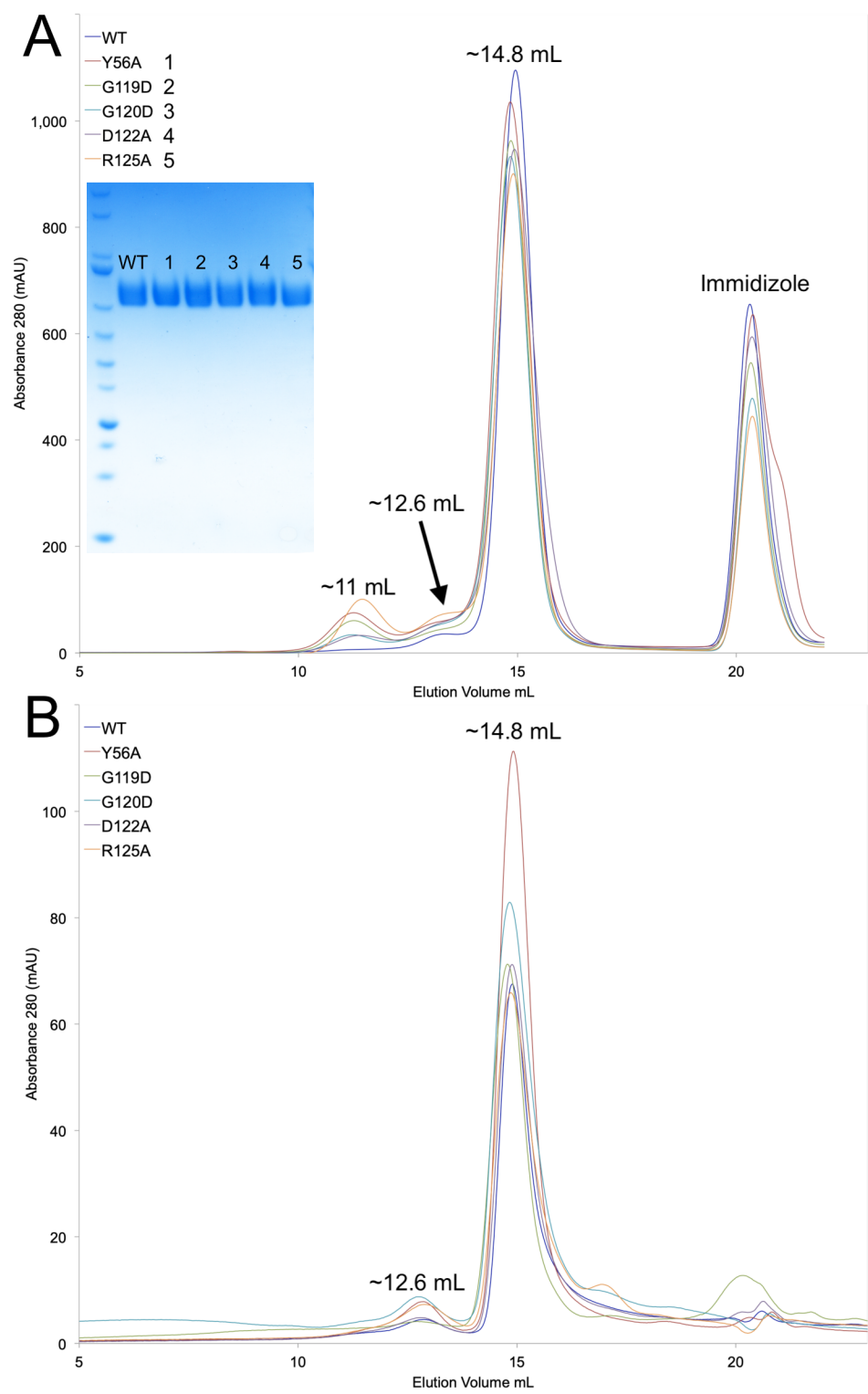

Supplement: S11 Fig — A) Analytical Size exclusion for fresh preparations of WT PD-L1, Y56A, G119D, G120D, D122A and R125A (inset of gel shows eluates off His60 resin) from 150mL of HEK suspension cells. VPA was added at 24hours, culture supernatants were collected on day 7 and purified over His60 resin. Nickel eluates were concentrated to 500uL and loaded onto the Superose 6 Increase column 10/300. The void for this column runs at 8.5mL. The peak containing dimer Fc-fusion is found ~14.8mL and was collected. There was also a small peak at ~11mL and a shoulder peak at ~ 13mL. B) Analytical Size exclusion was re-run 2 weeks later for WT PD-L1, Y56A, G119D, G120D, D122A and R125A. Eluates collected from the initial size exclusion run were stored at 4C for 2 weeks, concentrated 2X and 100uL was loaded onto the same analytical column. The predominant peak for all of the proteins remains around 14.8mL. There is no longer a peak at 11mL though a more defined peak is observed at ~13mL (previously where the shoulder peak was). This data suggests the PD-L1 Fc fusion protein is reasonably stable and the mutants show some deviation but behave very similarly to wild-type protein. (PDF) [file pone.0233578.s012.pdf]

Figure S12

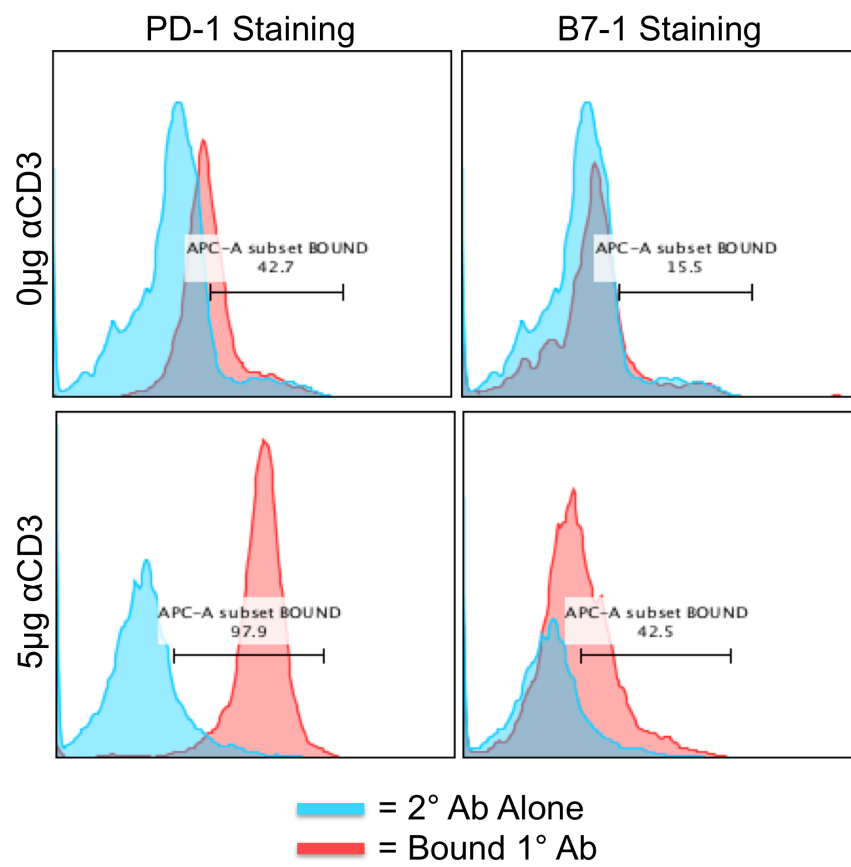

Supplement: S12 Fig — CD+ T-cells were isolated as described in the methods and plated in wells of a 96-well plates in the absence (TOP) or presence (BOTTOM) of 5ug anti-CD3. After 4-days cells were washed and incubated either with anti-human 647 secondary antibody alone (BLUE) or with primary anti-mPD-1 or anti-mB7-1 antibodies (human IgG1 from R&D Systems) as indicated. After staining cells were analyzed by flow cytometry and live 647 (APC) positive cells were gated based on the secondary antibody alone controls for each condition. (PDF) [file pone.0233578.s013.pdf]

Figure S13

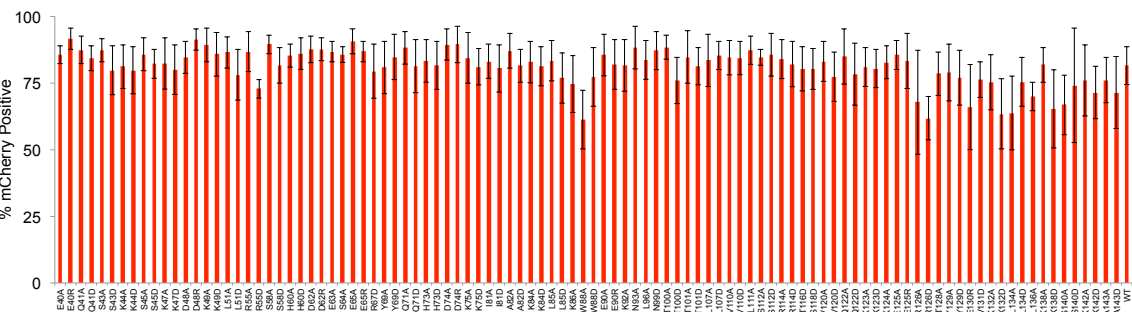

Supplement: S13 Fig — Graph shows the %mCherry positive HEK 293 cells transfected with wild-type mB7-1, mutant B7-1 or mCherry empty vector control. Data is the average from three independent transfections with error bars showing the standard deviation. (PDF) [file pone.0233578.s014.pdf]

Figure S14

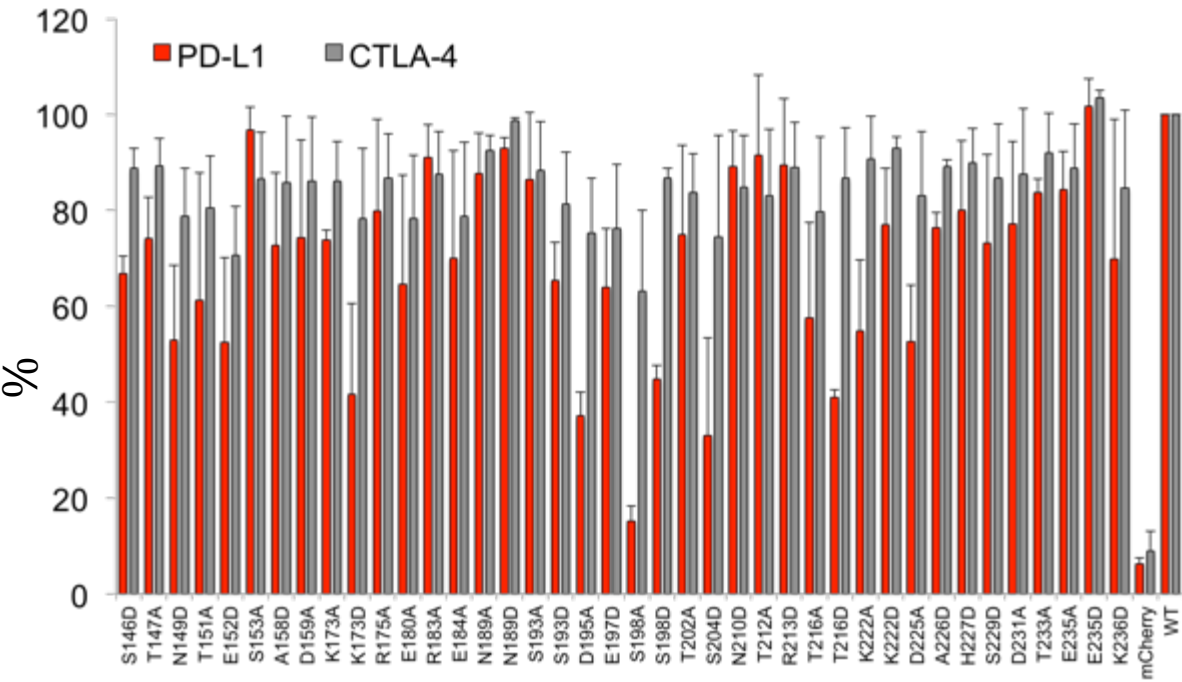

Supplement: S14 Fig — Graph highlighting the panel of 40 B7-1 IgC mutants examined for binding to mPD-L1 (Red Bars) and mCTLA-4 (Gray Bars) as described in the main text. Data shows percent bound from three independent experiments with error bars representing the standard deviation. (PDF) [file pone.0233578.s015.pdf]

Figure S15

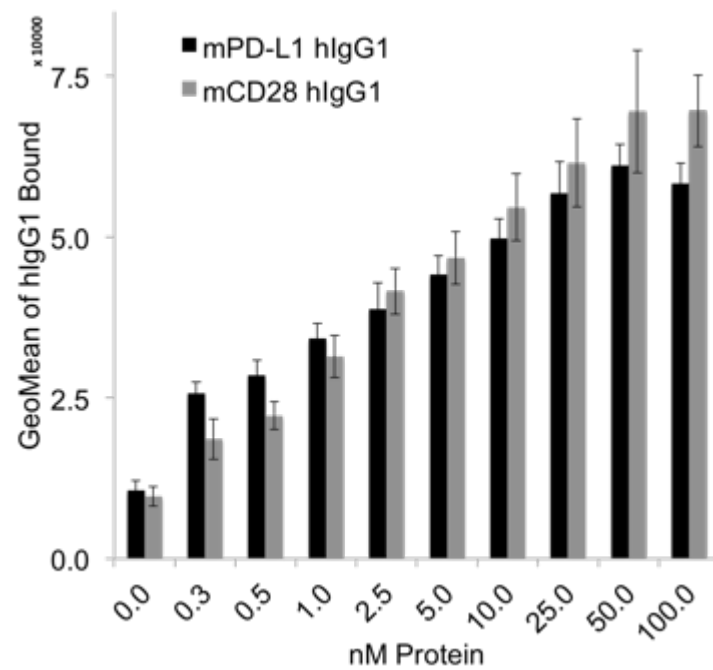

Supplement: S15 Fig — Data shows saturation binding curves used to determine the lowest concentration of either mPD-L1 hIgG1 or mCD28 hIgG1 protein to saturate protein A beads loaded with mB7-1 mIgG2a. For competition experiments, 5nM was added to B7-1 loaded beads in the presence of increasing concentrations of the competing proteins (mIgG2a control, CTLA-4 mIgG2a, CD28 mIgG2a, mPD-L1 mIgG2a). (PDF) [file pone.0233578.s016.pdf]

Figure S16

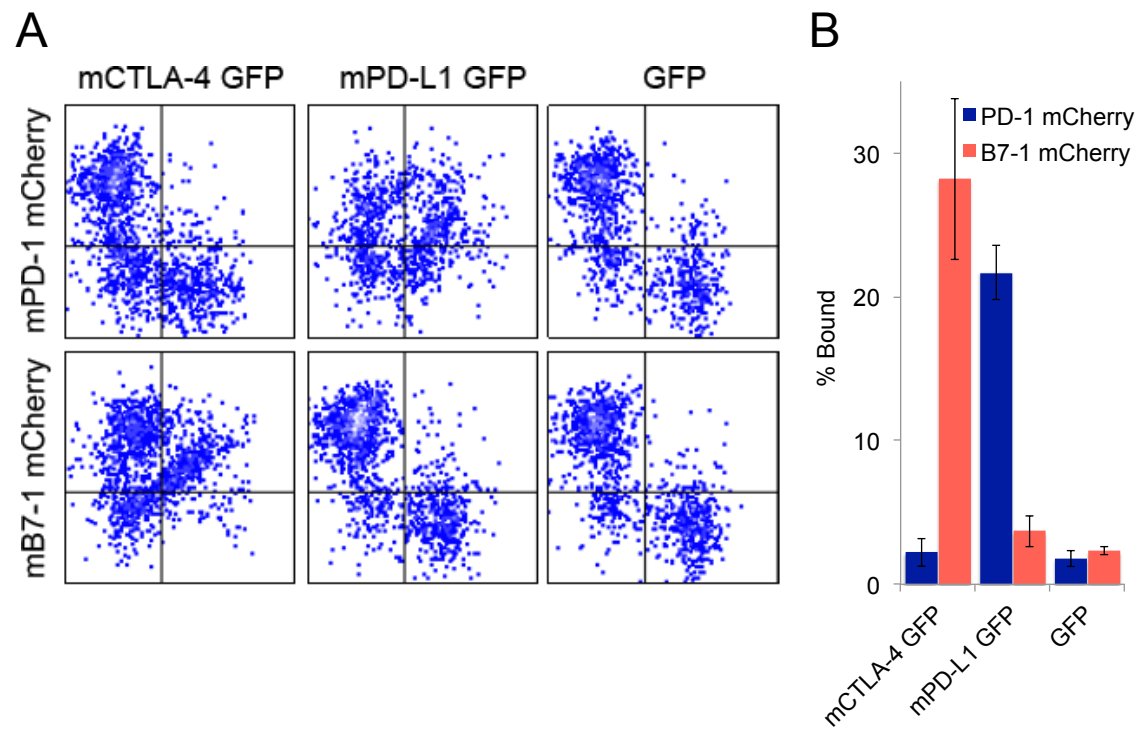

Supplement: S16 Fig — A) Representative scatter plots from cell-cell conjugation experiment. HEK 293 suspension cells expressing full-length mPD-1 and mB7-1 mCherry fusions were mixed 1:1 with cells expressing either mCTLA-4 GFP, mPD-L1 GFP or GFP control. Positive binding between the populations of cells is observed as an increase in the percentage of events in Q2 (upper right). B) Quantification of three independent cell-cell conjugation experiments. Data shows the average and standard deviation. (PDF) [file pone.0233578.s017.pdf]

Figure S17

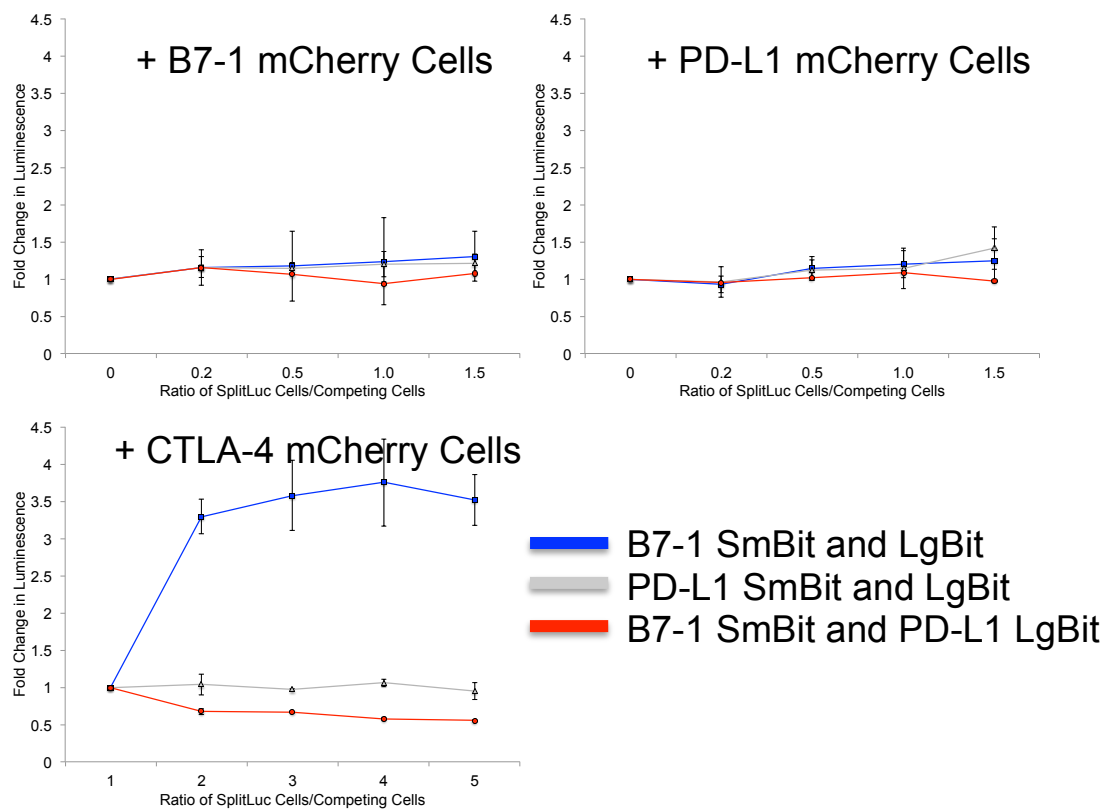

Supplement: S17 Fig — A) This experiment was setup similarly to that shown in Fig 7B. Cells expressing different SmBit/LgBit combinations as indicated were titrated with cells co-expressing B7-1 mCherry, PD-L1 GFP (solid lines) or with cells expressing CTLA-4 mCherry (dashed lines). No change in luminescence is observed with cells overexpressing B7-1 or PD-L1 suggesting the luminescence signal is not being driven by trans B7-1/PD-L1 binding. In contrast addition of CTLA-4 expressing cells results in a significant increase in B7-1 luminescence and a significant decrease in B7-1/PD-L1 cis luminescence. This is similar to what we demonstrate in the manuscript. The data represents three independent experiments. (PDF) [file pone.0233578.s018.pdf]
