# Supplementary material for: Mechanistic dissection of the PD-L1:B7-1 co-inhibitory immune complex
Source: PLoS One. 2020 Jun 4;15(6):e0233578. doi: 10.1371/journal.pone.0233578 (PMC7272049; doi:10.1371/journal.pone.0233578)
Supplement: S1 Table — The table shows scores for binding from the analysis of cell microarrays printed with PD-L1 mutants as shown in Fig 1E where “+” signifies a fluorescent intensity in the 647 channel (bound protein) comparable to WT, “−”signifies no detectable fluorescence and “R” signifies reduced fluorescent intensity compared to WT PD-L1. Scores reflect observations made from three independent experiments. Mutants labeled BLUE lost binding to both PD-1 and B7-1, those in RED lost binding only to B7-1 and those in GREEN lost binding only to PD-1. (PDF) [file pone.0233578.s019.pdf]

Table S1

	PD-1	B7-1
WT	+	+
mCherry	-	-
D49A	+	+
D49R	+	R
L53R	-	-
V54D	R	R
V54A	+	+
Y56A	+	-
Y56D	+	-
Q66A	+	+
Q66D	+	R
E72A	+	+
E72R	+	-
G119D	+	-
G119R	-	-
G120D	+	-
A121R	-	-
D122A	-	+
Y123A	-	+
Y123R	-	+
K124A	-	+
K124D	-	+
R125A	R	+
R125D	-	+
