# Supplementary material for: Mechanistic dissection of the PD-L1:B7-1 co-inhibitory immune complex
Source: PLoS One. 2020 Jun 4;15(6):e0233578. doi: 10.1371/journal.pone.0233578 (PMC7272049; doi:10.1371/journal.pone.0233578)
Supplement: S2 Table — The table shows the analysis of PD-1 and B7-1 binding to a subset of PD-L1 mutants as determined by flow cytometry as shown in Fig 2. Data is the calculated average and standard deviation from three independent experiments normalized to WT PD-L1 binding. Mutants labeled in RED lost binding only to B7-1 and those in GREEN lost binding only to PD-1. Mutants left black showed a reduction in both PD-1 and B7-1 binding. (PDF) [file pone.0233578.s020.pdf]

Table S2

	PD-1	B7-1
WT	1.00	1.00
mCherry	0.02 ± 0.02	0.10 ± 0.06
D26A	0.54 ± 0.26	0.93 ± 0.03
D26R	0.42 ± 0.07	0.96 ± 0.06
T37A	0.31 ± 0.03	0.26 ± 0.04
T37R	0.22 ± 0.11	0.17 ± 0.07
D49R	0.86 ± 0.06	0.31 ± 0.06
L53D	0.16 ± 0.10	0.09 ± 0.04
L53R	0.14 ± 0.10	0.10 ± 0.05
V54D	0.88 ± 0.12	0.30 ± 0.04
V54R	0.95 ± 0.04	0.49 ± 0.07
Y56A	1.07 ± 0.16	0.08 ± 0.04
Y56D	0.77 ± 0.10	0.07 ± 0.03
Y56R	0.64 ± 0.13	0.11 ± 0.06
Q66D	1.05 ± 0.07	0.54 ± 0.06
E72D	0.55 ± 0.07	0.56 ± 0.10
E72R	0.48 ± 0.15	0.11 ± 0.05
I115D	0.02 ± 0.01	0.05 ± 0.03
I116R	0.01 ± 0.01	0.01 ± 0.01
G119D	1.40 ± 0.20	0.08 ± 0.04
G119R	0.29 ± 0.10	0.02 ± 0.01
G120A	0.02 ± 0.01	0.01 ± 0.01
G120D	0.84 ± 0.20	0.05 ± 0.04
G120R	0.05 ± 0.03	0.04 ± 0.04
A121D	0.03 ± 0.03	0.03 ± 0.03
A121R	0.03 ± 0.03	0.03 ± 0.03
D122A	0.01 ± 0.01	0.74 ± 0.12
D122R	0.01 ± 0.01	0.01 ± 0.01
Y123A	0.01 ± 0.01	0.65 ± 0.08
Y123D	0.01 ± 0.01	0.33 ± 0.16
Y123R	0.01 ± 0.01	1.12 ± 0.04
K124A	0.02 ± 0.01	0.94 ± 0.06
K124D	0.03 ± 0.01	0.48 ± 0.05
K124R	0.02 ± 0.01	0.85 ± 0.08
R125A	0.03 ± 0.04	1.03 ± 0.02
R125D	0.02 ± 0.01	1.12 ± 0.08
