# Supplementary material for: Mechanistic dissection of the PD-L1:B7-1 co-inhibitory immune complex
Source: PLoS One. 2020 Jun 4;15(6):e0233578. doi: 10.1371/journal.pone.0233578 (PMC7272049; doi:10.1371/journal.pone.0233578)
Supplement: S3 Table — The table shows the analysis of B7-1 mutant binding to PD-L1, CTLA-4 and CD28 as determined by flow cytometry in Fig 4. Data shows the average and standard deviation from three experiments. Boxes highlighted RED show >20% binding comparable to WT B7-1 and boxes highlighted YELLOW show 20–50% binding comparable to WT B7-1. (PDF) [file pone.0233578.s021.pdf]

Table S3

	PD-L1	CTLA-4	CD28
WT	1.00	1.00	1.00
K44A	0.46 ± 0.02	0.96 ± 0.02	0.92 ± 0.05
K44D	0.01 ± 0.00	0.89 ± 0.04	0.47 ± 0.11
K47A	0.36 ± 0.02	0.97 ± 0.01	0.90 ± 0.05
K47D	0.02 ± 0.00	0.98 ± 0.01	0.94 ± 0.03
D48R	0.15 ± 0.05	0.82 ± 0.08	0.49 ± 0.07
K49A	0.03 ± 0.01	0.90 ± 0.09	0.90 ± 0.10
K49D	0.02 ± 0.02	0.93 ± 0.07	0.90 ± 0.07
R67D	0.73 ± 0.07	0.03 ± 0.02	0.02 ± 0.01
Y69A	0.56 ± 0.05	0.88 ± 0.02	0.09 ± 0.04
Y69D	0.47 ± 0.10	0.03 ± 0.02	0.00 ± 0.00
Q71A	0.80 ± 0.05	0.94 ± 0.03	0.03 ± 0.02
Q71D	0.22 ± 0.10	0.87 ± 0.04	0.01 ± 0.01
L85D	0.20 ± 0.07	0.97 ± 0.01	0.71 ± 0.11
W88A	0.82 ± 0.10	0.98 ± 0.01	0.57 ± 0.12
W88D	0.78 ± 0.03	0.97 ± 0.01	0.38 ± 0.02
N93A	0.19 ± 0.06	0.92 ± 0.06	0.87 ± 0.11
L96A	0.02 ± 0.01	0.95 ± 0.02	0.89 ± 0.10
T101A	0.28 ± 0.07	0.90 ± 0.04	0.69 ± 0.05
L107A	0.03 ± 0.02	0.90 ± 0.09	0.93 ± 0.03
L107D	0.02 ± 0.01	0.94 ± 0.06	0.93 ± 0.03
R114D	0.22 ± 0.06	0.94 ± 0.02	0.85 ± 0.08
S118D	0.09 ± 0.06	0.89 ± 0.08	0.48 ± 0.11
V120A	0.71 ± 0.06	0.62 ± 0.44	0.05 ± 0.02
V120D	0.09 ± 0.05	0.06 ± 0.05	0.01 ± 0.01
Q122A	0.82 ± 0.10	0.95 ± 0.03	0.28 ± 0.08
Q122D	0.25 ± 0.06	0.04 ± 0.04	0.01 ± 0.01
K123D	0.12 ± 0.05	0.92 ± 0.07	0.43 ± 0.08
Y129A	0.35 ± 0.05	0.88 ± 0.05	0.19 ± 0.01
Y129D	0.43 ± 0.06	0.73 ± 0.11	0.02 ± 0.01
V131D	0.64 ± 0.03	0.06 ± 0.00	0.01 ± 0.01
K132D	0.30 ± 0.06	0.93 ± 0.01	0.76 ± 0.04
L134D	0.51 ± 0.13	0.96 ± 0.02	0.29 ± 0.03
K142D	0.24 ± 0.09	0.93 ± 0.00	0.83 ± 0.00
K173D	0.41 ± 0.18	0.78 ± 0.14	N/D
D195A	0.37 ± 0.04	0.75 ± 0.11	N/D
S198A	0.15 ± 0.03	0.63 ± 0.16	N/D
S198D	0.44 ± 0.02	0.86 ± 0.02	N/D
S204D	0.33 ± 0.20	0.74 ± 0.21	N/D
T216D	0.40 ± 0.01	0.86 ± 0.10	N/D
